# The Resonance Raman Spectrum of Cytosine in Water: Analysis of the Effect of Specific Solute–Solvent Interactions and Non-Adiabatic Couplings

**DOI:** 10.3390/molecules28052286

**Published:** 2023-03-01

**Authors:** Qiushuang Xu, Yanli Liu, Meishan Wang, Javier Cerezo, Roberto Improta, Fabrizio Santoro

**Affiliations:** 1 School of Physics Engineering, Qufu Normal University, Qufu 273165, China; 2 School of Physics and Optoelectronics Engineering, Ludong University, Yantai 264025, China; 3 Consiglio Nazionale delle Ricerche, Istituto di Chimica dei Composti Organo Metallici (ICCOM-CNR), SS di Pisa, Area della Ricerca, Via G. Moruzzi 1, I-56124 Pisa, Italy; 4 Departamento de Química and Institute for Advanced Research in Chemical Sciences (IAdChem), Universidad Autónoma de Madrid, 28049 Madrid, Spain; 5 Consiglio Nazionale delle Ricerche, Istituto di Biostrutture e Bioimmagini (IBB-CNR), Via De Amicis 95, I-80145 Napoli, Italy

**Keywords:** vibrational Resonance Raman, vibronic effects, non-adiabatic couplings, solute–solvent interactions, DNA

## Abstract

In this contribution, we report a computational study of the vibrational Resonance Raman (vRR) spectra of cytosine in water, on the grounds of potential energy surfaces (PES) computed by time-dependent density functional theory (TD-DFT) and CAM-B3LYP and PBE0 functionals. Cytosine is interesting because it is characterized by several close-lying and coupled electronic states, challenging the approach commonly used to compute the vRR for systems where the excitation frequency is in quasi-resonance with a single state. We adopt two recently developed time-dependent approaches, based either on quantum dynamical numerical propagations of vibronic wavepackets on coupled PES or on analytical correlation functions for cases in which inter-state couplings were neglected. In this way, we compute the vRR spectra, considering the quasi-resonance with the eight lowest-energy excited states, disentangling the role of their inter-state couplings from the mere interference of their different contributions to the transition polarizability. We show that these effects are only moderate in the excitation energy range explored by experiments, where the spectral patterns can be rationalized from the simple analysis of displacements of the equilibrium positions along the different states. Conversely, at higher energies, interference and inter-state couplings play a major role, and the adoption of a fully non-adiabatic approach is strongly recommended. We also investigate the effect of specific solute–solvent interactions on the vRR spectra, by considering a cluster of cytosine, hydrogen-bonded by six water molecules, and embedded in a polarizable continuum. We show that their inclusion remarkably improves the agreement with the experiments, mainly altering the composition of the normal modes, in terms of internal valence coordinates. We also document cases, mostly for low-frequency modes, in which a cluster model is not sufficient, and more elaborate mixed quantum classical approaches, in explicit solvent models, need to be applied.

## 1. Introduction

Vibrational Resonance Raman (vRR) [1,2,3,4,5] spectroscopy has emerged as a very informative tool for the properties of molecular excited electronic states, and it has recently been profitably applied in many fields of biological and technological relevance [6,7,8,9,10,11,12].

When a molecule is irradiated, at an energy close to resonance with the transition energy between the ground state (GS) and a given excited state (ES), the Raman scattering is strongly enhanced. If the transition to the resonant state is bright, the intensity of the Raman bands depends on the Franck–Condon (FC) integrals between GS and ES vibrational states, and, for instance, the fundamental of a given mode increases with the structural change along it. Resonance Raman spectroscopy, therefore, delivers useful information on the main features of the ES involved, such as the equilibrium geometry, but also the force constants. In order to fully disclose the informative power of vRR experiments, it is often beneficial to compare them with computational predictions according to a suitable theoretical method [2]. A landmark in the simulations of vRR spectra was the contribution of Heller and colleagues and the so-called short-time approximation, best suited for the pre-excitation regime, where the intensity only depends on the excited state gradient and the normal mode frequency [5,13]. Since those pioneering works, several methods—too many to be reviewed here—have been introduced, to compute vRR in the true resonance regime, opening also the route to a detailed analysis of the dependence of vRR intensity on excitation wavelength—the so-called Raman excitation profiles. Among these approaches, it is worth mentioning those that, always grounded on harmonic approximation, account for the effects of frequency changes and Duschinsky mixings during the electronic transition, and for the role of Herzberg–Teller (HT) contributions, relevant for weak states [14,15,16,17,18,19,20,21]. Some of them are derived in a time-independent (TI), sum-over-states framework [17], whereas most of them adopt a time-dependent (TD) strategy, based on the Fourier transform of analytical correlation functions [14,15,16,18,19,20,21]. The latter ones are more efficient, but different correlation functions are needed for different types of transition: in most cases, derivations are limited to fundamental bands, although recently those for overtones and combination bands have been made available [22,23].

Many dyes of technological or biological relevance are characterized by an extended π system, conferring on them a rather dense ES manifold. For such systems, the idealization of a resonance with a single ES is often inadequate, and it is necessary to consider that several ES can be in quasi-resonance with the excitation wavelength, thus all contributing to the transition polarizability. In such a scenario, interference can occur, and although TI methods can be adopted [24], the effectiveness of TD approaches makes them more suited to facing large systems, many ES and/or large excitation ranges. Such computations including interference effects can now be performed, for instance, with the new release of our freely available code FCclasses 3.0 [23,25].

It is, however, easy to realize that, when different ES are close in energy, besides showing interference phenomena, they can also exhibit non-adiabatic couplings and/or even conical intersections [26]. In a recent contribution [27], we showed that the effect of interstate couplings on vRR spectra can also be included in semi-rigid systems such as thymine with seven coupled states and 39 normal modes, adopting a TD framework, resorting to non-adiabatic quantum-dynamical (QD) wave packet propagations, and exploiting the effectiveness of the multilayer (ML) generalization of the multiconfiguration time-dependent Hartree method (ML-MCTDH) [28,29,30]. More specifically, in order to describe different electronic states, we parameterized a Linear Vibronic Coupling (LVC) model, on the grounds of time-dependent density functional theory (TD-DFT) calculations [31,32], with a maximum-overlap diabatization approach. LVC models assume that inter-state couplings are linear functions of the normal coordinates, and that normal modes and frequencies of all coupled states coincide with the GS ones [33,34]. By this approach, we clearly showed that in thymine, considering interference effects but discarding inter-state couplings may lead to predictions even qualitatively wrong. At the same time, we showed that for this molecule couplings are so strong that they cannot be described within the perturbative HT regime, raising a general warning that, in similar situations, the straightforward application of the HT approach may lead to unphysical predictions [27].

**Figure 1:**
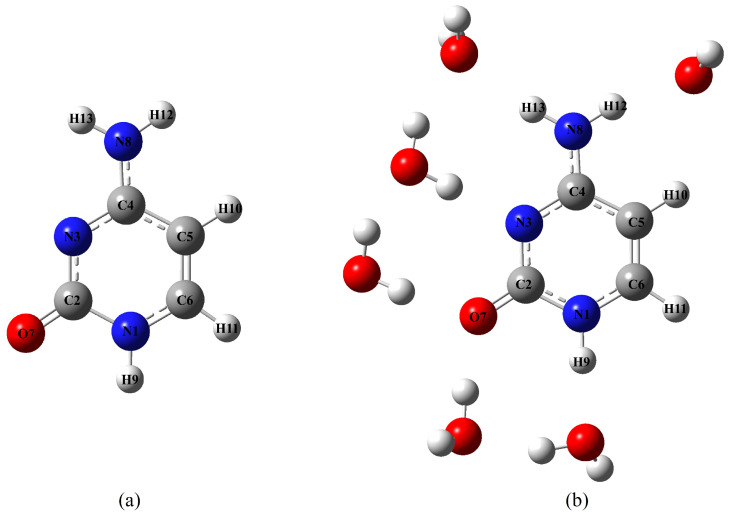
Molecular structures of the systems investigated in this work: (**a**) cytosine; (**b**) cluster cytosine· 6H${}_{2}$O.

In this contribution, we used our approach to simulate the vRR spectrum of cytosine (Figure 1) in water. Cytosine was an ideal example, because of the availability of a very rich set of experimental data reported in [35], and the existence of remarkable interstate couplings. Moreover, the study of the excited state dynamics of DNA and its constituents has attracted a lot of interest in recent decades [36,37,38,39], due to the biological and medical relevance of the photochemical processes triggered in DNA by UV absorption, which can lead to the damage of the genetic code [36]. Consequently, vRR has been widely adopted to investigate the structural dynamics of photoexcited nucleobases [35,40,41,42,43,44,45], nucleotides, [46], and oligonucleotides, [47,48], to cite some relevant papers. More recently, vRR has been used to characterize more complex DNA structures, and their interactions with therapeutic ligands [49,50,51,52,53].

Aside from obtaining additional information on the excited state properties of cytosine, we also investigated the effect of the solvent on the vRR spectra. To this end, we included the solvent effect in the vRR calculations with two different models. On the one hand, we resorted to an implicit solvation model, as the polarizable continuum model (PCM). On the other hand, we explicitly considered solute–solvent interactions, by studying at full quantum mechanical level a cluster model already adopted in the past [54], including six H_2_O molecules of the first solvation shell (right structure in Figure 1), using the PCM to account for bulk solvation effects. A similar cluster was also adopted by Mondal and Narayana [42], to simulate the vRR spectrum of Guanosine, which showed that placing one water molecule at each H-bond position is mandatory for correctly reproducing the effect of solvation on vRR spectra.

We computed the vRR spectra by two widely used hybrid functionals—PBE0 and the long-range corrected CAM-B3LYP functional—with the aim being to put our study on a more solid basis and, at the same time, to explore how the choice of the functional affected the simulated vRR.

## 2. Results

The lowest-energy ES for cytosine in water at the FC position are listed in Table 1, and for the cluster with six water molecules in Table 2. In agreement with previous computational studies [31,55,56], independently of the computational model, three bright $\pi \pi^{*}$ transitions fell below 6.5 eV, with several close-lying n$\pi^{*}$ and Rydberg ($Ry_{\sigma }$) transitions. Assignments were done based on Natural Transition Orbitals (NTOs), as shown in Section S1 of the Supplementary Materials, where we also report the Kohn–Sham Molecular Orbitals (MOs). The lowest energy n$\pi^{*}$ states involved the Lone Pair of the N3 (n${}_{N}$) and O7 (n${}_{O}$) atoms, significantly coupled (see [31] for a detailed discussion). The two functionals provided a similar description of the FC region, with PBE0 bright states being slightly red-shifted. Solute–solvent hydrogen bonds (Table 2) stabilized $\pi \pi_{3}^{*}$, whereas they have been confirmed [55] to destabilize $\pi \pi_{1}^{*}$ and, especially, n$\pi^{*}$ and Rydberg states.

### 2.1. Absorption Spectra

**Figure 2:**
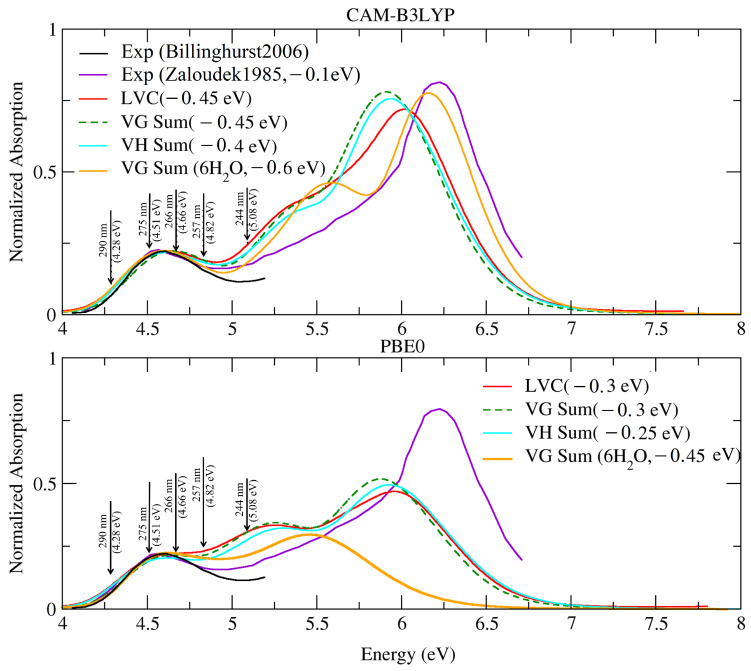
Absorption spectra of cytosine—computed by the LVC model, FC∣VG, FC∣VH, and FC∣VG—which, considering the 6H${}_{2}$O effect, convoluted with a Gaussian of HWHM = 0.12 eV and a Lorentzian of HWHM = 0.04 eV. Experimental data, in water, from [35] (black) and [57] (purple). Arrows indicate the excitation wavelength used in the vRR experiments in [35]. For better comparison with the experiments, all spectra were renormalized so as to have the same maximum intensity for the lowest peak. Experimental spectra in [57] are given in (dm${}^{3}$ mol${}^{-1}$ cm${}^{-1}$), and absolute intensities are compared in Figure S5 in the Supplementary Materials, confirming a good agreement.

Figure 2 compares the ABS spectra computed in water, adopting different vibronic models (see section Materials and Methods for definition)., to two sets of experimental spectra. The first one was taken from [35], the same source of the vRR spectra, and was limited to the lowest energy band. The arrows denote the excitation wavelengths used to measure the vRR spectra: all of them were resonant with the first absorption band, except the highest-energy one, at 244 nm, which fell in a valley between two bands that, according to our calculations, were associated with $\pi \pi_{1}^{*}$ and $\pi \pi_{2}^{*}$. The second experimental spectrum was extracted from a figure in [57], and encompassed a much larger energy range; therefore our extraction of the data was made with presumably smaller precision: this inaccuracy may explain the apparent shift between the two experimental spectra (see also Section S2 of the Supplementary Materials).

As discussed in detail in the Supplementary Materials, the shape of the computed spectrum nicely agreed with that measured below 6.5 eV, reported in [57]. From the quantitative point of view, all the computed spectra were, almost uniformly, blue-shifted by 0.4∼0.6 eV (CAM-B3LYP), and by 0.25∼0.45 eV (PBE0), with respect to the experimental one, depending on the adopted computational model. Comparison of the VH and VG spectra suggested that the effect of the Duschinsky rotation and frequency changes was small, apart from a slight improvement in the position of the lowest energy band (∼0.05 eV); however, as discussed in Section S2 of the Supplementary Materials, the estimate of such a shift may have been biased by the existence of imaginary frequencies for the ES. The PBE0 spectra were closer to the experimental one, for what concerned the position of the lowest energy band, but CAM-B3LYP reproduced better the relative energy and position of the different peaks. In particular, PBE0 failed to reproduce the height of the highest peak: however, this error was only due to the existence of intruder states with CT character, making it necessary to increase the number of excited states computed to ’reach’ the bright excited state responsible for that band. In fact, the inclusion of state S${}_{10}$ with E${}_{gf}$ = 6.68 eV and $\delta_{OPA}$ = 0.60 would allow for also reproducing such a band. On balance, the CAM-B3LYP predictions for the cluster cytosine·6H${}_{2}$O in the PCM were in very good agreement with the experiments, as far as the relative position and the eight of the first and third bands were concerned. It is worth noting, however, that such an agreement does not imply that all the possible effects played by the water molecules of the bulk were fully accounted for by the PCM. In particular, we recall that we needed to add a Gaussian broadening with HWHM = 0.12 eV to phenomenologically recover the effect of the fluctuation of the whole solvent surrounding the cytosine. Moreover, with cytosine·6H${}_{2}$O, we also observed a slight increase of the overestimation of the relative intensity of the central peak, at ∼5.5 eV, an effect that may have been related to an intrinsic deficiency of the static cluster model that we were considering. In any case, as all the theoretical models provided a fairly reliable description of the first absorption band, in the following section we present our analysis of the vRR spectra predicted by all of them.

### 2.2. Vibrational Resonance Raman Spectra in the Excitation Range Measured in the Experiments

We started our analysis of the computed vRR spectra by focusing on the range of excitation frequencies investigated in the experiments, from 290 to 244 nm [35]. As a first step, we analyzed the effect of interference and non-adiabatic couplings on the spectra, by comparing the predictions of the three different protocols, **VG*****Sum***, **VG*****Int***, and LVC (see section Materials and Methods for definition).

**Figure 3:**
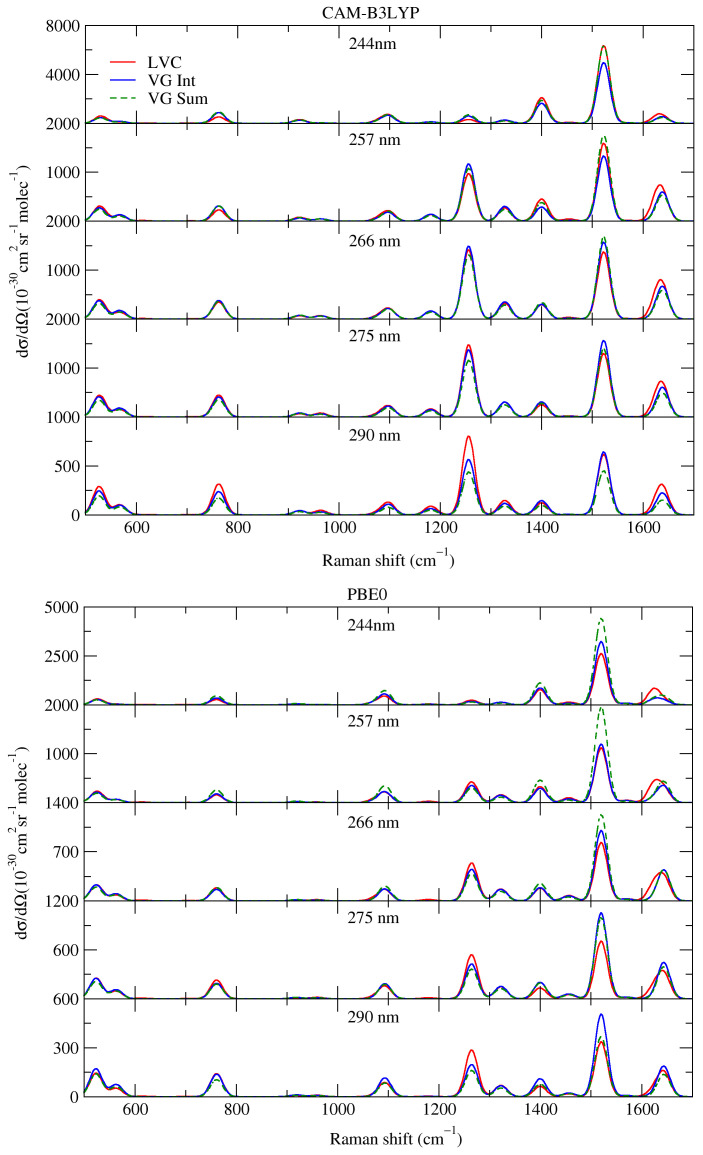
Vibrational Resonance Raman spectra of cytosine, computed by the LVC model (red lines), FC|**VG*****Int*** (blue lines), and FC|**VG*****Sum*** (dashed green lines) levels, convoluted with a Lorentzian with damping, γ = 0.04 eV, and a Gaussian of HWHM = 0.12 eV, calculated by CAM-B3LYP and PBE0 with 6-311G+(d,p) basis sets in water.

Figure 3 shows that the three sets of spectra were quite similar; therefore, unlike what happened for shorter excitation wavelengths (see following sections), in the 290–244 energy range, the effects of interference and of non-adiabatic couplings were generally weak. A closer inspection, however, reveals that interference and inter-state couplings both introduced moderate differences. In some cases—as for the ∼1250 cm${}^{-1}$ band at 290 nm in the CAM-B3LYP spectra—**VG*****Sum*** and **VG*****Int*** were very similar, and moderately different from LVC, indicating that interference was negligible, whereas non-adiabatic effects had some impact on the intensity. Conversely, for the ∼1500 cm${}^{-1}$ band at 244 nm in the PBE0 spectra, **VG*****Int*** was similar to LVC, and slightly different from **VG*****Sum***, suggesting that, in this case, the interference between the close-lying states was more important than their couplings. Interestingly, for the same band, CAM-B3LYP provided a different picture as, in that case, LVC was very similar to **VG*****Sum***, and moderately different from **VG*****Int***, suggesting that considering only interference may introduce differences that are not confirmed by a more complete approach, such as LVC. These conflicting indications confirmed the conclusions we reached in [27], i.e., that in a situation with many close-lying states, in principle, the most robust predictions for vRR are provided by LVC non-adiabatic calculations. Nonetheless, we further stress that in the specific case of cytosine, and the energy range investigated in the experiments, the non-adiabatic effects were not large.

In the second step of our analysis, the effects of frequency changes and Duschinsky mixing were monitored by comparing vRR predictions in VH and VG models. As shown in the Supplementary Materials, such effects played a very minor role. In summary, in the following discussion, we were able to confidently use the ’computationally cheap’ **VG*****Int*** approach, as the reference method for our comparison with the experimental vRR spectra.

#### 2.2.1. Comparison with Experiments

**Figure 4:**
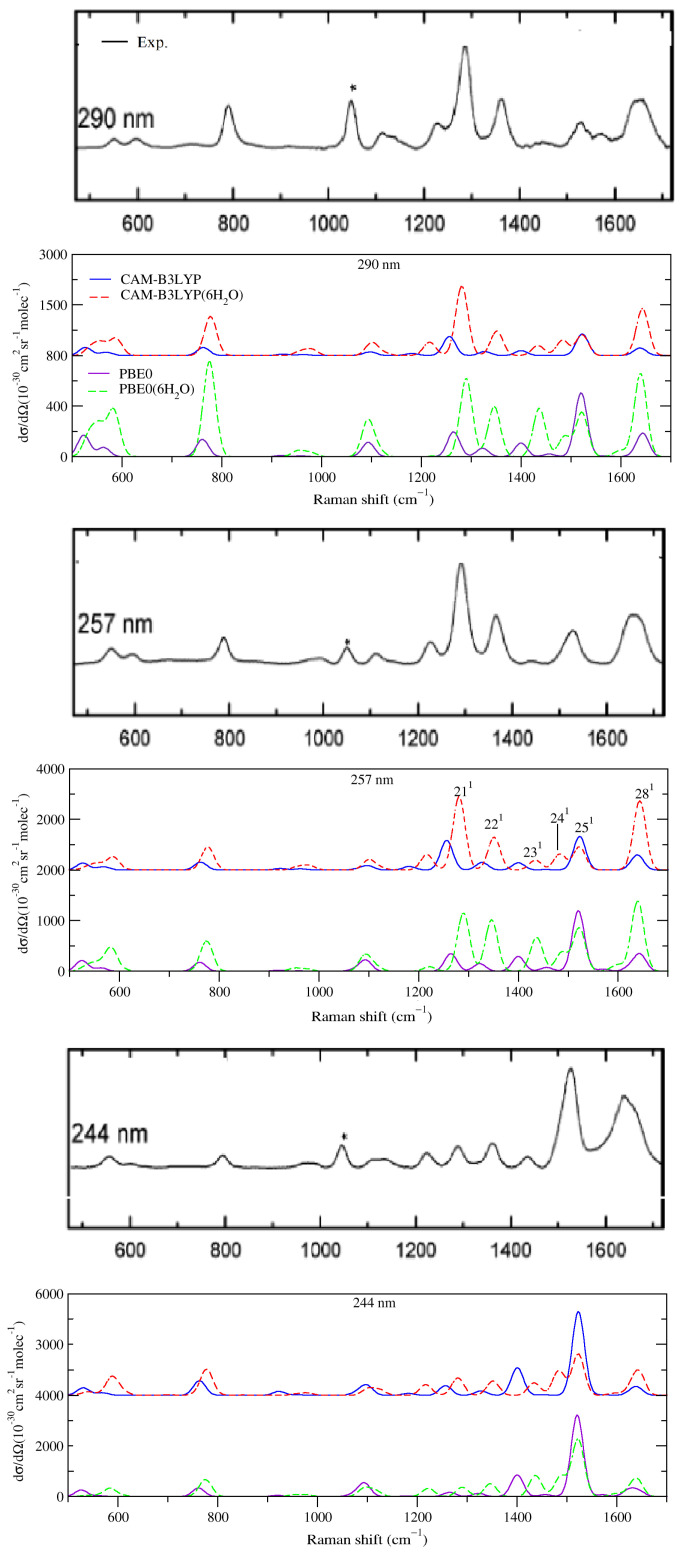
Vibrational Resonance Raman spectra, computed by **VG*****Int***, for cytosine in water (PCM) and the cluster cytosine·6H${}_{2}$O in the PCM, convoluted with a Lorentzian with damping, γ = 0.04 eV, and a Gaussian with HWHM = 0.12 eV, calculated by CAM-B3LYP and PBE0 and 6-311G+(d,p) basis set. The black, thick panels report the experimental data in aqueous solutions; reprinted with permission of [35]. Copyright 2007 American Chemical Society. The experimental band marked with an asterisk is attributed to the internal standard.

We computed the vRR spectra for the five excitation wavelengths explored in the experiments [35]: 290; 275; 266; 257; and 244 nm. However, according to both the experiments and the computations, the shapes of the spectra at 266 and 275 nm were similar to those at 290 and 257 nm. As a consequence, we reported the former two in the Supplementary Materials (Figure S6). We started discussing the spectra obtained without considering explicit solvent molecules (Figure 4).

The spectra recorded at 257 and 290 nm were very similar, and this feature was well reproduced by CAM-B3LYP, which described fairly well the main peaks and relative intensities up to 1400 cm${}^{-1}$. In particular, we found a very intense peak at 1256 cm${}^{-1}$ (21${}^{1}$) and, on its right, two weaker bands at 1328 cm${}^{-1}$ (assigned to the fundamental of mode 22, 22${}^{1}$) and 1400 cm${}^{-1}$ (23${}^{1}$). The largest discrepancy, with respect to the experiments, was observed at >1400 cm${}^{-1}$, where two peaks, with intensity ∼50% smaller than the main features at ∼1300 cm${}^{-1}$, were found at ∼1500 and ∼1650 cm${}^{-1}$ in the experiments. These peaks were predicted also by CAM-B3LYP, 25${}^{1}$ and 28${}^{1}$, but their intensity was significantly overestimated, with the former as intense as the peak 21${}^{1}$.

On balance, the vRR spectra provided by PBE0 computations (bottom panel in Figure 4), though in good qualitative agreement with the experiments, were less accurate than the CAM-B3LYP ones: for example, the intensity of the peak at 1550 cm${}^{-1}$ further increased and dominated the spectrum.

Decreasing the excitation wavelength at 244 nm had a dramatic effect on the vRR experimental spectra. Indeed, the band at 1300 cm${}^{-1}$ became much weaker, whereas the intensity of the two bands at ∼1550 and 1650 cm${}^{-1}$ increased remarkably, the former becoming the most intense. These changes were nicely captured by CAM-B3LYP (and PBE0), which also reproduced the large decrease in the intensity of the 1300 cm${}^{-1}$ peak. On balance, the CAM-B3LYP vRR spectrum was in good agreement with the experimental ones, except for a strong underestimation of the intensity of the band at 1650 cm${}^{-1}$.

#### 2.2.2. Effect of Explicit Solvent on vRR

Computation of the vRR spectra for the cluster cytosine·6H${}_{2}$O shed light on the effect of specific solute–solvent interactions on the vRR spectra. As shown in Figure 4, for CAM-B3LYP (top) and PBE0 (bottom), when the hydrogen bonds with the water molecules of the first solvation shell were considered in our calculations, the general agreement with the experiments remarkably improved. In particular, in the vRR spectra at 257 and 290 nm, the intensity of the peak 28${}^{1}$ at ∼1650 cm${}^{-1}$ significantly increased and, at the same time, that of 25${}^{1}$ decreased. On balance, the vRR spectral lineshape was well-reproduced, for what concerned both the position and the relative intensity of the different peaks.

The computed 244 nm spectrum was also in good agreement with the experimental ones. In particular, both functionals correctly predicted that the two most prominent peaks fell at ∼1550 cm${}^{-1}$ and at ∼1620 cm${}^{-1}$, with the former being the most intense, and the inclusion of specific solute–solvent interactions remarkably improved the prediction of their relative height.

However, in some particular cases the adoption of the cluster model introduced larger discrepancies with the experiments: this was the case with the intensity of the two bands <800 cm${}^{-1}$ at 244 nm, which was remarkably overestimated for cytosine·6H${}_{2}$O. A detailed analysis (Section S3.4.3 of the Supplementary Materials) suggests that this outcome depended on a combination of different effects. On the one hand, the existence of water molecules removed the C${}_{s}$ symmetry, making out-of-plane modes vRR-active (in particular, mode 11 at 776 cm${}^{-1}$). In the gas phase, the energy profile along these modes would show either a true minimum in C${}_{s}$ or a double-well, whereas in solution it was perturbed by the fluctuating positions of the water molecules. On the other hand, the increase of the intensity of the band at ∼600 cm${}^{-1}$ was due to a remarkable increase in the displacement along some in-plane ring distortions that moved atoms, such as O, involved in H-bonds. In both cases, the observed discrepancies with the experiments suggested that a single cluster (with an “optimized structure”) was not a good model for such situations. Moreover, for these low-frequency modes, anharmonic effects, neglected here, were probably quite substantial.

A second issue with the cluster model occurred at all excitation wavelengths, and was due to the appearance of a new computed band at ∼1475 cm${}^{-1}$, assigned as 24${}^{1}$, which was absent (or weak) in the experimental spectra. Such a discrepancy may have been due to an overestimation of the frequency differences between modes 24 and 25. It should also be noted that, whereas the cluster model could introduce some effects of the specific solute–solvent interaction, it did not account for the inhomogeneous broadening of the vibrational frequencies due to solvent fluctuations. Such an effect could lead to the computed bands collapsing into a single one, in better agreement with the experiments.

Our results, therefore, confirm the necessity of further methodological advancements, for a robust description of the solvent effects on vRR spectra. To achieve this goal, we consider very promising the adoption of mixed quantum classical models, where the solvent is described by classical molecular dynamics, whereas the solute vibrations retain their quantum nature. In this scenario, we note the remarkable progress achieved during the last years in this field by Gómez et al. [58,59,60], who also adopted polarizable force fields that accounted for the mutual solute/solvent polarization. The hybrid scheme adiabatic MD generalized Vertical Hessian (Ad-MD|gVH) we introduced few years ago, Ref. [61] is also suitable for these applications: indeed, Gómez et al. [53] recently adopted a modified version of it, to study vRR spectra of Doxorubicin, both in solvent and intercalated into DNA. Concretely, these authors coupled the Ad-MD|gVH strategy with a clustering analysis, documenting that this approach was especially suitable for reproducing vRR spectra of the system embedded in both environments, with simulated spectra in very good agreement with the experiments.

Before concluding this section, we note that, by adopting the cluster model, the PBE0 results also improved, as can be seen by looking at the relative intensities of bands 22${}^{1}$, 23${}^{1}$, 25${}^{1}$, and 28${}^{1}$. However, even in this case, PBE0 was not able to reproduce the experimental result, which, with the peak at ∼1300 cm${}^{-1}$, corresponding to 21${}^{1}$, was most intense at 290 and 257 nm. Moreover, when compared to CAM-B3LYP, the relative errors on the intensities of bands 22${}^{1}$, 23${}^{1}$, and of band 28${}^{1}$ at 244 nm, were larger.

#### 2.2.3. Further Analysis of the Modes Involved in the Most Intense Transitions

**Figure 5:**
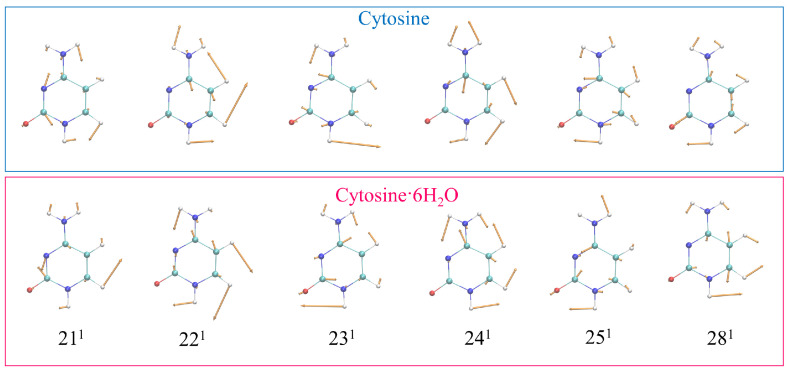
Schematic representation of the most relevant vRR-active vibrational modes of cytosine in the high-frequency range, and cytosine·6H${}_{2}$O, removing the components of the 6H${}_{2}$O from the Hessian, calculated by CAM-B3LYP and the 6-311G+(d,p) basis sets in water.

The analysis described in the previous section showed that the most intense experimental bands bands could be assigned to the fundamentals 21${}^{1}$, 22${}^{1}$, 23${}^{1}$, 24${}^{1}$, 25${}^{1}$, and 28${}^{1}$. In order to get a more in-depth interpretation of the spectra, we focused on the CAM-B3LYP results, which better compared with the experiments, and in Table 3 we list the most relevant modes together with their (scaled) frequencies and dimensionless shifts Δ (the displacement of the S${}_{1}$ and S${}_{2}$ geometry with respect to the GS one). Their Cartesian representations, with arrows, are given in Figure 5, whereas their assignments, in terms of internal valence coordinates, provided by FCclasses, are in Table S5 in the Supplementary Materials (Table S7 for PBE0). We recall that the normal modes for the cluster were computed after removing the components of the coordinates of the water molecules in the Hessian and, therefore, they were strictly localized on the cytosine. In the Supplementary Materials, we show that even including such contributions, both the modes (Figure S7) and the vRR spectra (Figure S11) were quite similar.

At 290 and 257 nm, the contribution of the resonance with S${}_{1}$ was clearly dominant, and the relative vRR intensities of the fundamental bands were directly connected to the square of the displacements Δ of S${}_{1}$ along the corresponding mode (Δ1). The largest value of Δ1 along mode 21 was consistent with the fact that 21${}^{1}$ was the most intense vRR band. The additional higher-frequency bands were much weaker than 21${}^{1}$. The rise of the extra 24${}^{1}$ band with the cluster model can be explained by the Δ1 along this mode, predicted when including the hydrogen bond effects (0.41), being much larger than in the PCM only (0.09). As for the two highest-frequency bands, the fact that 25${}^{1}$ was much more intense than 28${}^{1}$ was due to the larger displacement (0.91 vs. −0.51). However, when the effects of the 6 H${}_{2}$O were considered, the predicted displacements along these two modes became more similar, improving the comparison with the experiments. On the same basis, the increase of band 22${}^{1}$, with the cluster model, was due to the increase of the displacement (from 0.43 to 0.54) along this mode. These findings confirm the large sensitivity of the vRR intensities to the displacements. It is interesting to note that, in a simple pre-resonance (or short-time) approximation, the relative intensities depended on $\omega^{2}\Delta^{2}$ [17]. According to this simple rule, from the data in Table 3, the ratio of the intensities of 22${}^{1}$, with respect to the highest peak 21${}^{1}$, varied from 21% to 38%, explaining most of the observed differences in Figure 4.

Further analysis in the Supplementary Materials shows that the presence of the water molecules did not significantly change the displacement of the internal coordinates between the GS and S${}_{1}$ minima: this suggests that their effect on the vRR spectra was mostly due to the fact that they changed the contribution of the most displaced valence coordinates to the different normal modes. For instance, the better agreement with the experiments obtained with the cluster model can be traced back to the fact that this model predicted a smaller contribution of the stretching C4C5 to mode 25, and a larger contribution of the stretching C6C5 to mode 28.

An analogous analysis for the two bands at 600 and 800 cm${}^{-1}$ is reported in Section S3.4 of the Supplementary Materials.

When exciting at 244 nm, the contributions of the S${}_{1}$ and S${}_{2}$ states became comparable, and the shape of the vRR spectrum changed remarkably. Inspection of Figure 3 (and Figure S8 in the Supplementary Materials for the cluster cytosine·6H${}_{2}$O) indicates that, although interference and non-adiabatic couplings did play a role, the effect of the excitation wavelength was already reproduced by the simple **VG*****Sum*** approach. Since, in the latter model, the contributions of the two states were considered independently, and were only summed, this result can be understood from the simple analysis of the displacements Δ on S${}_{1}$ and S${}_{2}$ (Δ1 and Δ2 in Table 3). The displacements along mode 25 and 28 increased and decreased remarkably in S${}_{2}$, with respect to S${}_{1}$, explaining the inversion of the relative intensities in the experiments. Such change was large in the PCM, and more moderate for the cluster, with 6H${}_{2}$O, in line with the better agreement with the experiments. On the same basis, the displacement along mode 21 decreased steeply, and this fact, together with the smaller frequency of this mode, explains why at 244 nm the relative intensity of 21${}^{1}$, with respect to 25${}^{1}$, was reduced drastically.

### 2.3. Raman Excitation Profiles

**Figure 6:**
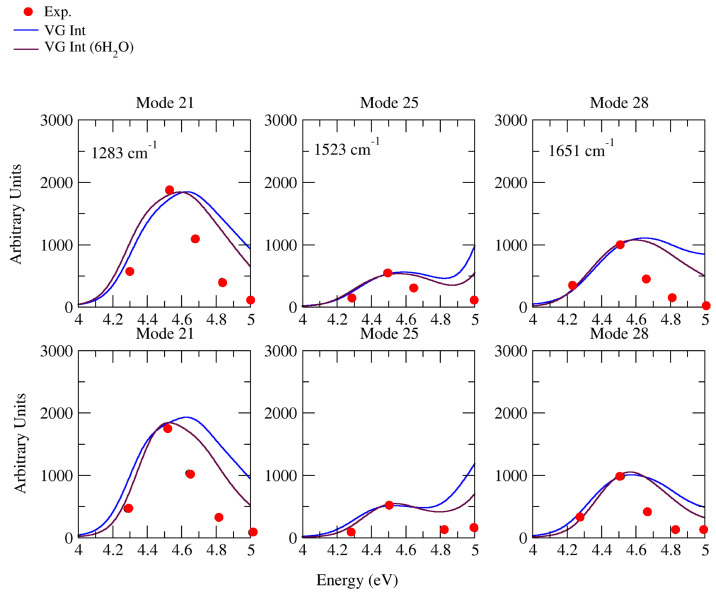
Raman excitation profiles of the four modes of cytosine, computed at **VG*****Int*** and **VG*****Int***, considering the effects of 6H${}_{2}$O, convoluted with a Lorentzian with damping γ = 0.04 eV and a Gaussian of HWHM = 0.12 eV, calculated by CAM-B3LYP (**top**) and PBE0 (**below**) with the 6-311G+(d,p) basis sets in water. Experimental frequencies are out of parentheses; our computational frequencies are in parentheses. Intensity is renormalized and our computational wavelength of **VG*****Int*** is blue-shifted by 3629 cm${}^{-1}$ (0.45 eV); **VG*****Int*** considering the effects of 6H${}_{2}$O is blue-shifted by 4839 cm${}^{-1}$ (0.6 eV) for CAM-B3LYP (**top**); **VG*****Int*** are blue-shifted by 3629 cm${}^{-1}$ (0.45 eV); **VG*****Int***, considering the effects of 6H${}_{2}$O, is blue-shifted by 4839 cm${}^{-1}$ (0.6 eV) for PBE0 (**below**), to match the experiment.

In Figure 6, we provide a synoptic picture, comparing the experimental Raman excitation profiles at 1283 cm${}^{-1}$, 1523 cm${}^{-1}$, and 1651 cm${}^{-1}$, in the range 4–5 eV [35], with the profiles computed by CAM-B3LYP (top) and PBE0 (bottom) for the corresponding fundamental bands 21${}^{1}$, 25${}^{1}$, and 28${}^{1}$. The agreement was generally good, apart from a general progressive overestimation of the relative vRR intensity for excitation frequencies > 4.5 eV. Such a discrepancy was probably connected to the overestimation of the computed absorption intensity (see Figure 2), with respect to the experimental one [35], at λ < 260 nm (energy > 4.77 eV), which becomes more evident in the valley between the two lowest bands, at ∼5 eV. This interpretation is supported by the better agreement found in the excitation profile of the 6H${}_{2}$O model, for which the absorption intensity in that region was smaller.

**Figure 7:**
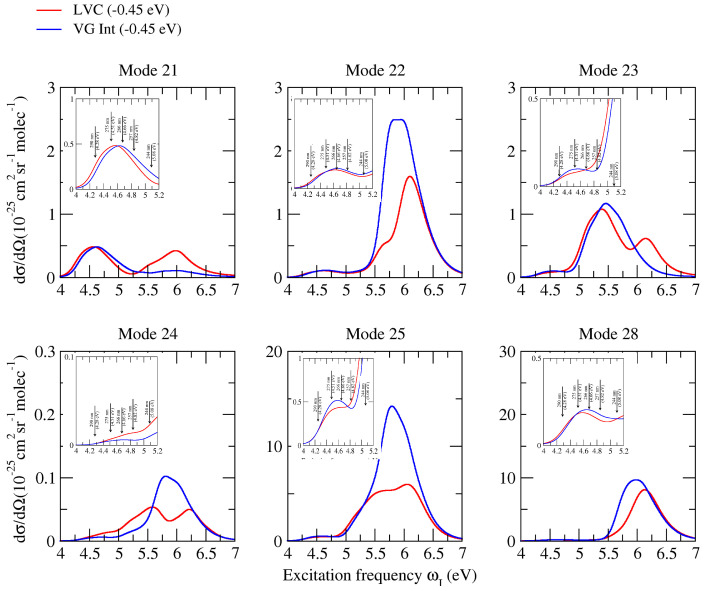
Raman excitation profiles of the six modes of cytosine, computed at non-adiabatic LVC level, and **VG*****Int*** convoluted with a Lorentzian with damping γ = 0.04 eV and a Gaussian of HWHM = 0.12 eV, calculated by CAM-B3LYP and the 6-311G+(d,p) basis sets in water.

**Figure 8:**
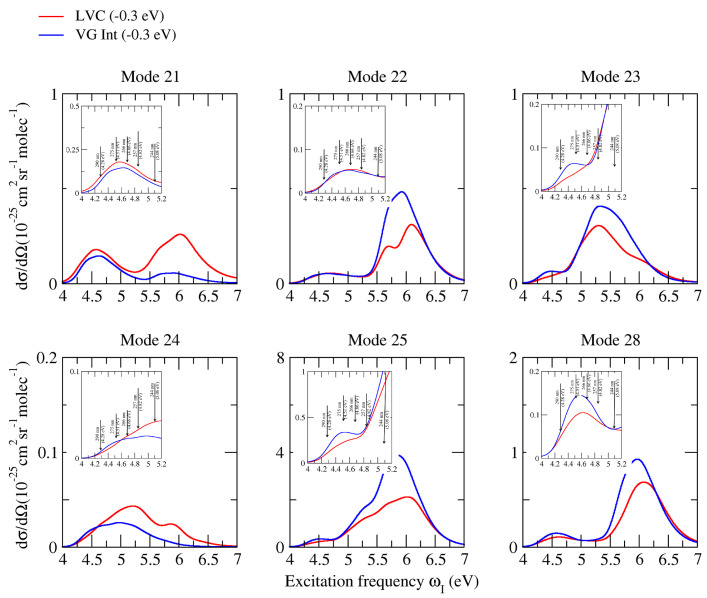
Raman excitation profiles of the four modes of cytosine, computed at non-adiabatic LVC level, and **VG*****Int*** convoluted with a Lorentzian with damping γ = 0.04 eV and a Gaussian of HWHM = 0.12 eV, calculated by PBE0 and the 6-311G+(d,p) basis sets in water.

Whereas, as already observed in Figure 3, non-adiabatic effects were very moderate for excitations resonant with the first absorption band (corresponding to the experimental measures), the inspection of the Raman excitation profiles in a broader excitation range can allow a much more complete analysis of the possible role played by these couplings on vRR. To this end, Figure 7 (CAM-B3LYP) and Figure 8 (PBE0) consider the six most important high-frequency modes already discussed in the previous sections, and report their computed Raman excitation profiles up to an excitation of 7 eV, covering the possible resonance with all the first absorption bands. Inspection of the figures shows that, according to both functionals, the difference between the prediction of LVC and **VG*****Int*** models became very remarkable at excitation energies > 5.5 eV (the peak of the second absorption band), leading in some cases to halving the predicted intensity (check bands 22${}^{1}$ and 25${}^{1}$ at ∼6 eV). For the band 24${}^{1}$, such an effect became relevant even at 5 eV (the valley between the first two absorption bands). These findings indicate that, for all bands, the coupling between ${\pi \pi^{*}}_{2}$ and ${\pi \pi^{*}}_{3}$ plays a major role in vRR intensity, in a way that is not reproduced by simply accounting from the interference of their contribution to the transition polarizability (also considered by **VG*****Int***). This outcome indicates that the adoption of a non-adiabatic LVC model is fundamental to the investigation of the vRR at these energies and, more generally, strongly recommended when large energy ranges are considered, as in these cases the occurrence of remarkable non-adiabatic interactions is more likely.

## 3. Conclusions

In this contribution, we presented a computational study of the vibrational Resonance Raman spectra of cytosine in water, described either with the PCM or with a cluster with six H-bonded water molecules in the PCM, and by using two different density functionals, i.e., CAM-B3LYP and PBE0. We included in our computations the first eight excited states, and the distinguished effects of interference and inter-state couplings were disentangled by comparing the predictions of three different protocols (LVC, **VG*****Int***, and **VG*****Sum***). In this way, we were able to simulate the spectra in a large interval of excitation frequencies, although a special focus was devoted to five different excitation wavelengths, from 290 nm and 244 nm, explored in the experiments in [35]. With our computations we showed that, in this spectral region, interference and non-adiabatic effects were very moderate, and that the vRR intensities were always dominated by the resonance with the first bright state, except at 244 nm, in the valley between the two lowest-energy absorption bands, where the resonance with the second bright state $\pi \pi_{2}^{*}$ also played a major role. As a consequence, when exciting at 244 nm, the shape of the vRR spectrum changed remarkably, and such changes could be clearly interpreted by looking at the different displacement of the equilibrium structure of $\pi \pi_{1}^{*}$ and $\pi \pi_{2}^{*}$, with respect to the ground state. At excitation energies higher than those explored experimentally (λ< 244 nm), corresponding with the resonance with $\pi \pi_{2}^{*}$ and $\pi \pi_{3}^{*}$, non-adiabatic couplings had a larger impact on the vRR spectra, and their effect was different from that of simple interference between the contribution of different states to the transition polarizability. This finding confirmed the conclusions of [27], i.e., that, in general, for systems with many quasi-degenerate states, the adoption of LVC models is strongly recommended.

Both PBE0 and CAM-B3LYP provided vRR spectra in good agreement with the experimental ones, though the latter appeared more accurate. In previous studies, [31,56] we have shown that these two functionals give a slightly different description of the interplay between bright and low-energy dark n$\pi^{*}$ states, whose stability is overestimated by PBE0. The comparison with the experimental vRR in this contribution indicates that PBE0 also provides slightly less accurate geometries of the bright excited states. This result shows that relatively small differences in the description of the excited states in the FC region, which have no significant impact on the absorption spectra, are much more evident in vibrational spectroscopies like vRR, confirming that this spectroscopy is also very useful for validating the accuracy of different computational methods for electronic excited states.

Adoption of the cluster model cytosine·6H${}_{2}$O allowed for introducing the effect of specific solute–solvent interactions, leading, in general, to a remarkable improvement of the comparison with the experiments, especially for high-frequency modes. As the displacements of bond lengths and angles were only slightly perturbed by the presence of the solvent molecules, we concluded that the improved agreement with the experiments was mainly due to a modification of the composition of the normal modes, in terms of valence internal coordinates. Interestingly, this finding is in line with previous results reported for the simulation of vRR spectra of guanosine computed by short-time approximation, and neglecting interferential effects between different states, ref. [42] which show that the effect of explicit solvation on vRR spectra arises from the modulation of normal modes, rather than to induce changes on the gradient (displacements). In a few cases, however–in particular, for two modes < 800 cm${}^{-1}$–the inclusion of explicit water molecules worsened the agreement with the experiments, causing an overestimation of the vRR intensity. We believe that this finding was likely due to the anharmonic nature of such modes. In particular, in one case, we could document that the discrepancy was due to the fact that explicit water molecules removed the C${}_{s}$ symmetry, making non-planar modes active for vRR. Their energy profile, however, was well-known to be anharmonic, with a possible double well shape, and depended on the instantaneous position of the molecules, so that a static cluster model could not provide an accurate description of their motion. In order to go beyond this model, it will be necessary to adopt mixed quantum classical approaches, allowing a dynamical description of a large shell of solvent molecules. On the other hand, in this contribution, we clearly evidenced the necessity to address the effect of inter-state couplings for dyes with many close-lying states, such as nucleobases, with the adoption of LVC models. Ad-MD|gVH has been recently extended, to deal with coupled-states systems, Ad-MD|gLVC, and applied to absorption spectroscopy [62]: its possible further extension to vRR, to account simultaneously for the effects of inter-state couplings and explicit solvent models, will be explored in the near future.

## 4. Materials and Methods

### 4.1. Theory

Let us consider a molecule with a ground electronic state *g* and a manifold of excited states *m* potentially relevant to the vRR signal. The key quantity by which to compute the vRR spectra from the ground vibrational state $\nu_{0}$ to the final vibrational state $\nu_{f}$, both associated with the electronic state *g*, consists of the Cartesian components (ρ,σ=x,y,z) of the transition polarizability tensor $\alpha_{\rho \sigma }^{f0}$, which, in a TD formalism, can be written as

(1) $$\alpha_{\rho \sigma }^{f0}\left(\omega_{I}\right)=\frac{i}{\hbar }\sum_{m,p}\int_{0}^{\infty }dte^{i(E_{g0}/\hbar +\omega_{I})t-\gamma t}\chi_{\rho \sigma }^{f0,mp}\left(t\right)$$

In Equation (1), $\omega_{I}$ is the excitation frequency, $E_{g0}$ is the energy of the initial state, and γ is the lifetime of the excited states, considered equal. The correlation functions $\chi_{\rho \sigma }^{f0,mp}\left(t\right)$ have the following expression:

(2) $$\chi_{\rho \sigma }^{f0,mp}\left(t\right)=\langle m;v_{f}|\mu_{\rho }^{gm}e^{-iHt/\hbar }\mu_{\sigma }^{gp}|p;v_{0}\rangle$$

where $\mu_{\rho }^{gm}$ is the ρ Cartesian component of the electronic transition dipole element between states *g* and *m*. Once the transition polarizability is computed, the vRR intensity can be evaluated as follows [2,17]:

(3) $$I\left(\pi /2,{⊥}^{s}+{\|}^{s},{⊥}^{I}\right)=\frac{\omega_{S}^{4}I}{16\epsilon_{0}^{2}c_{0}^{4}\pi^{2}}\frac{45a^{2}+7g^{2}+5d^{2}}{45}$$

where the rotational invariants, *a*, $g^{2}$, and $d^{2}$, are:

(4) $$\begin{array}{ccc} a & = & \frac{\alpha_{xx}^{f0}+\alpha_{yy}^{f0}+\alpha_{zz}^{f0}}{3}\\ g^{2} & = & \frac{1}{2}\left(|\alpha_{xx}^{f0}-\alpha_{yy}^{f0}{|}^{2}+|\alpha_{xx}^{f0}-\alpha_{zz}^{f0}{|}^{2}+{\left|\alpha_{yy}^{f0}-\alpha_{zz}^{f0}\right|}^{2}\right)+\\ & & \frac{3}{4}\left(|\alpha_{xy}^{f0}+\alpha_{yx}^{f0}{|}^{2}+|\alpha_{xz}^{f0}+\alpha_{zx}^{f0}{|}^{2}+|\alpha_{yz}^{f0}+\alpha_{zy}^{f0}{|}^{2})\right)\\ d^{2} & = & \frac{3}{4}\left(|\alpha_{xy}^{f0}-\alpha_{yx}^{f0}{|}^{2}+|\alpha_{xz}^{f0}-\alpha_{zx}^{f0}{|}^{2}+|\alpha_{yz}^{f0}-\alpha_{zy}^{f0}{|}^{2})\right) \end{array}$$

In practice, the correlation functions in Equation (2) arise from the propagation of wavepackets started in any of the bright electronic states, *m*, and those with m≠p are different from zero only if inter-state couplings exist. It is worth noting that even in cases where such couplings vanish, the transition polarizability $\alpha_{\rho \sigma }^{f0}\left(\omega_{I}\right)$ is the sum of contributions over the resonant electronic states *m*, and, since such a sum is taken before computing the rotational invariants, interferential effects can occur.

### 4.2. Analytical Correlation Functions for Vibrational Resonant Raman Spectroscopy for “Single-State” Harmonic Systems

For systems in which neither the initial *g* nor the quasi-resonant *m* states are involved in significant non-adiabatic couplings, the transition polarizability in Equation (1) can be obtained by summing *m* independent correlation functions, one for each resonant state (m=p). If all states *m* are characterized by harmonic potential energy surfaces (PES), the correlation functions $\chi_{\rho \sigma }^{f0,mm}\left(t\right)$ have an analytical expression, dependent on the parameters of the *g* and *m* PES and their transition dipole. The expressions implemented in our FCclasses 3.0 code were reported in [23,27]. They include the possibility that the normal modes of the ground and excited states are different, and are generally related by a Duschinsky transformation:

(5) $$Q_{g}=JQ_{m}+K$$

In general, the elements $\mu_{\rho }^{gm}$ and $\mu_{\sigma }^{mg}$ depend on nuclear coordinates, and are expanded as a Taylor series, in terms of one set of normal coordinates (here, we choose $Q_{g}$)

(6) $$\mu_{\rho }^{gm}\left(Q\right)=\mu_{\rho }^{gm}\left(Q_{0}\right)+\sum_{k=1}^{N_{vib}}\frac{\partial \mu_{\rho }^{gm}\left(Q\right)}{\partial Q_{kg}}Q_{kg}+\cdot \cdot$$

The zero-order constant term is known as Franck–Condon (FC), whereas, according to first-order perturbation theory, the linear terms—known as Herzberg–Teller (HT)—introduce the effects of the inter-state couplings induced by displacements along the normal coordinates. Such an approach is perturbative, and is very convenient for describing the borrowing of intensity for a weak electronic state from a coupled bright state well-separated in energy [63]. However, application of the HT approach to cases where couplings are strong, and lead to significant mixings of the vibronic states, can lead to large artefacts, as has been shown for absorption [64] and circular dichroism [65] and, more recently, for vRR spectroscopy [27].

### 4.3. Numerical Computation of the Correlation Functions for Coupled States

For systems in which the manifold of the quasi-resonant states *m* is involved in significant inter-state couplings, the correlation functions in Equation (2) must be computed numerically. To that end, it is useful to introduce a set of diabatic states $|d_{n}\rangle$ and the associated LVC Hamiltonian:

(7) $$\begin{array}{ccc} H & = & \sum_{i}\left(T+V_{nn}^{dia}\left(q\right)\right)|d_{n}\rangle \langle d_{n}|\\ & & +\sum_{n,r>n}V_{nr}^{dia}\left(q\right)\left(|d_{n}\rangle \langle d_{p}|+|d_{p}\rangle \langle d_{n}|\right) \end{array}$$

where now, for convenience, we use as coordinates the set of the dimensionless normal coordinates of the ground state $q=\omega^{1/2}Q_{g}$ and the associated momenta p.

The kinetic (*T*) and potential (*V*) terms are

(8) $$\begin{array}{ccc} T & = & \frac{1}{2}p^{T}\Omega p \end{array}$$

(9) $$\begin{array}{ccc} V_{nn}^{dia}\left(q\right) & = & E_{i}^{0}+\lambda_{nn}^{T}q+\frac{1}{2}q^{T}\Omega q, \end{array}$$

(10) $$\begin{array}{ccc} V_{nr}^{dia}\left(q\right) & = & \lambda_{nr}^{T}q. \end{array}$$

where Ω is the diagonal matrix of the vibrational frequencies of state *g*; therefore, $V_{nn}^{dia}\left(q\right)$, the PES of diabatic state *n*, is a quadratic function of q that shares the same normal modes and frequencies of *g*, and the inter-state couplings $V_{nr}^{dia}\left(q\right)$ are linear functions of q.

Diabatic states, $d_{n}$, are obtained through a transformation of the adiabatic states. They are defined so as to be ideally independent of the normal coordinates; therefore, their transition dipole with the ground state is expected to be approximately constant, and we can safely consider only the zero-order FC term in Equation (6).

### 4.4. Computation Details

Electronic calculations were performed with the Gaussian16 package of programs [66], adopting the Density Functional Theory (DFT) level for GS properties and the TD-DFT for ES ones. Two different functionals, CAM-B3LYP and PBE0, were used in combination with the 6-311G+(d,p) basis set. PBE0 is a standard hybrid functional, with a quality generally comparable to the more popular B3LYP, to describe excited state properties [67,68], but with the appealing property that it does not use any adjustable parameters [69,70]. Conversely, CAM-B3LYP [71] is a long-range corrected functional, which provides a balanced description of different kinds of electronic states—such as valence excitations, charge-transfers and Rydberg transitions [72]—that more often occur when large ranges of excitation energies are of interest, as in the present contribution. Cytosine minimum in the GS belongs to the $C_{s}$ symmetry point group, as we verified by checking that all the computed vibrational frequencies were real. Vibronic computations neglecting inter-state couplings, and exploiting analytical time-correlation functions, were performed with version 3.0 of FCclasses [23,25] (freely available). On the other hand, in order to evaluate the impact of inter-state couplings, we resorted to QD simulations by the ML-MCTDH method [29,30], as implemented in the Quantics code [73,74]: in that case, we adopted an LVC model, parameterized with a maximum-overlap diabatization protocol implemented in the freely distributed Overdia code [31,75].

We focused on the 8 lowest excited electronic states, comprising the lowest-lying bright states and the close-lying dark states. For the FCclasses calculations—i.e., those neglecting inter-state couplings—we employed Vertical Gradient (VG) and Vertical Hessian (VH) harmonic models. Both approaches expand the GS and ES PES around the GS equilibrium geometry, but they differentiate, in as much as VG assumes that final ES states share the same normal modes and frequencies of the GS, while VH recomputes the Hessian of each ES, thus allowing for the inclusion of frequency changes and Duschinsky mixing effects [76]. For the ML-MCTDH method, we used a variable mean field (VMF) with a Runge–Kutta integrator of order 5 and accuracy $10^{-7}$, for both absorption (ABS) and vRR spectra.

The effect of water on the GS and ES properties was taken into account, using the PCM with the linear response implementation [77,78]. To investigate the effect of specific solute–-solvent interactions on the ABS and vRR spectra, we also considered the cluster cytosine· 6H${}_{2}$O embedded in the PCM, a model already exploited to study the electronic spectroscopy of cytosine in water in [54], and depicted in Figure 1. It is well known that clusters of a solute, even with few solvent molecules, can exhibit several stable conformations [79,80]. In order to investigate qualitatively the effects of H-bonds on the spectroscopy of cytosine, we decided to compare the predictions of the simple chromophore in the PCM with those for a cluster in which all of its H-bonds capabilities were saturated. In practice, we placed a water molecule donating an H to each of the lone pairs of the carbonyl and of the nitrogen (3 molecules), and a water molecule accepting an H from each of the NH bonds (3 molecules). After checking that the steric hindrance was reasonable, we optimized the molecular structure in the GS, obtaining a true minimum (all positive frequencies) belonging to the C${}_{1}$ symmetry point group. In a previous study, we showed that, when bulk solvent effects are described with the PCM, including a small number of water molecules of the first solvation shell can quite satisfactorily simulate the solvent shifts on the absorption spectrum of cytosine derivatives [81].

For this latter system, we adopted only the VG model with the TD analytical implementation in FCclasses. The attempt to describe the motion of the solvent molecules of the cluster with harmonic models can give rise to artefacts [82]. On the other hand, in the bulk, the position and orientation of the water molecules of the first solvation shell are expected to fluctuate, a phenomenon that cannot be reproduced with a cluster model. Therefore, in order to avoid having results too affected by the specific static position of the 6 water molecules, we defined effective normal modes on the solute only, at fixed solvent molecules, removing from the Hessian the terms corresponding to their Cartesian coordinates. These effective normal modes, however, felt the presence of the water molecules, allowing for the capture of the effects of the mutual solute/solvent polarization on the solute vibrations, which have been included by other authors employing polarizable force fields [58,83]. In any case, as noted in the Supplementary Materials, we found that the results for the vRR spectra were extremely similar to those obtained by diagonalizing the full Hessian of the cluster, i.e., allowing the normal modes to spread on both the solute and the solvent molecules.

Both homogeneous and inhomogeneous contributions to broadening were included in the ABS and vRR spectra. For ABS, we simply applied a Voigt profile, with a Gaussian component with HWHM = 0.12 eV, and a Lorentzian with γ = 0.04 eV. For the vRR spectra, the procedure had to be performed in two steps [35]: firstly, the homogeneous contribution was included in the evaluation of the transition polarizability, with γ = 0.04 eV, and the vRR spectra were computed; secondly, such narrow vRR excitation profiles were convoluted with a Gaussian broadening, with HWHM = 0.12 eV. The value of γ for the homogeneous broadening 0.04 eV was taken to be very similar to the one estimated in [35], to simulate dephasing, whereas the Gaussian HWHM = 0.12 eV was estimated so that the computed ABS spectra approximately matched the width of the experimental ones. The vRR spectra were computed as a 2D function of the Raman shift $\omega_{I}$–$\omega_{S}$ and the incident frequency $\omega_{I}$. The computations delivered vRR spectra that were stick lines along the Raman shift, and so they were further convoluted along this coordinate with a Gaussian lineshape (HWHM = 15 cm${}^{-1}$). It should be noted that inhomogeneous solvent effects can be addressed non-phenomenologically, by adopting explicit solvent models: in combination with vibronic computations, this has been done for different spectroscopies [61], including vRR [58,59].

For a fair comparison of computed and experimental vRR, GS vibrational frequencies (Raman shift) were scaled by a typical factor 0.96, to correct for the inaccuracy of the adopted electronic structure theory, and for the lack of anharmonic effects. Moreover, in order to reproduce the same resonance condition in the experiment, the computed excitation frequencies were shifted with respect to the experimental ones, by the difference of the maxima of the calculated and experimental ABS spectra [24].

When considering more than one quasi-resonant electronic state, in order to disentangle the effect of interferences and inter-state couplings, we computed the vRR intensities, by using three different protocols:

- **LVC**—included both the effects of the inter-state couplings and of the interference of the different states; computations were performed with Overdia (LVC parameterization) and Quantics (QD), plus local scripts, to combine the correlation functions;
- **VG*Int*** —neglected the effects of inter-state couplings, but considered the interference of the different states, i.e., a sum was taken in Equation (1), over all the relevant states *m* (p=m); computations were performed with FCclasses (in Section S4 of the Supplementary Materials, we show that ML-MCTDH computations with Quantics delivered identical spectra);
- **V*Sum*** —neglected both the effects of inter-state couplings and interferences; the vRR intensity was thus simply the sum of what was obtained by repeating the computation *n* times, considering only one ES state *m* in Equation (1) (p=m); afterwards, the vRR intensities in Equation (3) were summed for all relevant states; computations were performed with FCclasses.

## Figures and Tables

**Table 1:** Symmetry, vertical excitation energies E${}_{gf}$ (eV), and oscillator strengths ($\delta_{OPA}$) of the first nine excited states for cytosine ($C_{s}$ symmetry point group), calculated by CAM-B3LYP and PBE0, with the 6-311+G(d,p) basis sets in water (PCM).

**STATE**	**CAM-B3LYP**			
	**Sym.**	**E_*gf*_(eV)**	***δ*_OPA_**	**Descr.**
S${}_{1}$ ($\pi \pi_{1}^{*}$)	A′	5.15	0.12	$\pi_{H}\pi_{L}^{*}$
S${}_{2}$ (n$\pi_{1}^{*}$)	A″	5.70	0.0028	n${}_{N}$$\pi_{L}^{*}$
S${}_{3}$ ($\pi \pi_{2}^{*}$)	A′	5.94	0.21	$\pi_{-1}\pi_{L}^{*}$
S${}_{4}$ (${Ry}_{1}$)	A″	6.25	0.0054	$\pi_{H}Ry_{\sigma 1}$
S${}_{5}$ (n$\pi_{2}^{*}$)	A″	6.30	0.0007	n${}_{O}$$\pi_{+1}^{*}$+n${}_{O}$$\pi_{L}^{*}$ +
S${}_{6}$ ($\pi \pi_{3}^{*}$)	A′	6.48	0.38	$\pi_{H}\pi_{+1}^{*}$
S${}_{7}$ (n$\pi_{3}^{*}$)	A″	6.57	0.0002	n${}_{O}$$\pi_{L}^{*}$-n${}_{O}$$\pi_{+1}^{*}$
S${}_{8}$ (${Ry}_{2}$)	A″	6.71	0.0038	$\pi_{-1}Ry_{\sigma 2}$
S${}_{9}$ ($\pi \pi_{4}^{*}$)	A′	6.89	0.40	$\pi_{-1}\pi_{+1}^{*}$
**STATE**	**PBE0**			
	**Sym.**	**E_*gf*_(eV)**	***δ*_OPA_**	**Descr.**
S${}_{1}$ ($\pi \pi_{1}^{*}$)	A′	4.98	0.092	$\pi_{H}\pi_{L}^{*}$
S${}_{2}$ (n$\pi_{1}^{*}$)	A″	5.44	0.0025	n${}_{N}$$\pi_{L}^{*}$ +n${}_{O}$$\pi_{L}^{*}$
S${}_{3}$ ($\pi \pi_{2}^{*}$)	A′	5.65	0.15	$\pi_{-1}\pi_{L}^{*}$
S${}_{4}$ (n$\pi_{2}^{*}$)	A″	5.78	0.0002	n${}_{O}$$\pi_{L}^{*}$-n${}_{N}$$\pi_{L}^{*}$
S${}_{5}$ (${Ry}_{1}$)	A″	6.13	0.0053	$\pi_{H}Ry_{\sigma 1}$
S${}_{6}$ (n$\pi_{3}^{*}$)	A″	6.17	0.0001	n${}_{O}$$\pi_{+1}^{*}$
S${}_{7}$ ($\pi \pi_{3}^{*}$)	A′	6.30	0.21	$\pi_{H}\pi_{+1}^{*}$
S${}_{8}$ (${Ry}_{2}$)	A″	6.62	0.0028	$\pi_{-1}Ry_{\sigma 2}$
S${}_{9}$ (n$\pi_{4}^{*}$)	A″	6.66	0.0025	n${}_{N}$$\pi_{+1}^{*}$

**Table 2:** Vertical excitation energies E${}_{gf}$ (eV) and oscillator strengths ($\delta_{OPA}$) of the first nine excited states for cytosine·6H${}_{2}$O ($C_{1}$ symmetry point group) in water, calculated by CAM-B3LYP and PBE0, with the 6-311+G(d,p) basis sets in water.

**STATE**	**CAM-B3LYP**		
	**E_*gf*_(eV)**	***δ*_OPA_**	**Char.**
S${}_{1}$ ($\pi \pi_{1}^{*}$)	5.25	0.19	$\pi_{H}\pi_{L}^{*}$
S${}_{2}$ ($\pi \pi_{2}^{*}$)	5.98	0.15	$\pi_{-1}\pi_{L}^{*}$
S${}_{3}$ (n$\pi_{1}$)	6.07	0.028	n${}_{N}$$\pi_{L}^{*}$ +n${}_{O}$$\pi_{L}^{*}$
S${}_{4}$ ($\pi \pi_{3}^{*}$)	6.31	0.27	$\pi_{H}\pi_{+1}^{*}$
S${}_{5}$ (${Ry}_{1}$)	6.47	0.0054	$\pi_{H}Ry_{\sigma 1}$
S${}_{6}$ (n$\pi_{2}$)	6.71	0.0043	n${}_{O}$$\pi_{+1}^{*}$ +n${}_{N}$$\pi_{+1}^{*}$
S${}_{7}$ ($\pi \pi_{4}^{*}$)	6.84	0.54	$\pi_{-1}\pi_{+1}^{*}$
S${}_{8}$ (${Ry}_{2}$)	6.96	0.0075	$\pi_{-1}Ry_{\sigma 2}$
S${}_{9}$ (n$\pi_{3}$)	6.97	0.025	n${}_{O}$$\pi_{+1}^{*}$ -n${}_{N}$$\pi_{+1}^{*}$
**STATE**	**PBE0**		
	**E_*gf*_(eV)**	***δ*_OPA_**	**Char.**
S${}_{1}$ ($\pi \pi_{1}^{*}$)	5.11	0.15	$\pi_{H}\pi_{L}^{*}$
S${}_{2}$ ($\pi \pi_{2}^{*}$)	5.69	0.11	$\pi_{-1}\pi_{L}^{*}$
S${}_{3}$ (n$\pi_{1}$)	5.78	0.022	n${}_{N}\pi_{L}^{*}$ +n${}_{O}\pi_{L}^{*}$
S${}_{4}$ ($\pi \pi_{3}^{*}$)	6.08	0.16	$\pi_{H}\pi_{+1}^{*}$
S${}_{5}$ (${Ry}_{1}$)	6.22	0.0028	$\pi_{H}Ry_{\sigma 1}$
S${}_{6}$ (n$\pi_{2}$)	6.26	0.0001	n${}_{O}\pi_{L}^{*}$ +n${}_{N}\pi_{L}^{*}$
S${}_{7}$ (n$\pi_{3}$)	6.48	0.0013	n${}_{N}\pi_{+1}^{*}$ +n${}_{O}\pi_{+1}^{*}$
S${}_{8}$ (CT)	6.54	0.0048	CT
S${}_{9}$ (${Ry}_{3}$)	6.64	0.0054	$\pi_{H}Ry_{\sigma 2}$

**Table 3:** Analysis of the modes responsible for the strongest vRR fundamental bands in resonance with the S${}_{1}$ electronic state. Frequencies (ω) scaled by 0.96 are in wavenumbers, whereas Δ1 and Δ2 are the dimensionless shifts of the equilibrium geometry in S${}_{1}$ and S${}_{2}$, respectively.

**Mode**	**CAM-B3LYP**					
	**Cytosine**			**Cytosine·6H_2_O**		
	***ω***	**Δ1**	**Δ2**	***ω***	**Δ1**	**Δ2**
21	1256	0.98	0.27	1281	−0.92	−0.42
22	1327	−0.43	0.13	1351	0.54	−0.15
23	1400	−0.43	−0.80	1433	−0.33	−0.49
24	1454	0.09	−0.07	1483	0.41	0.69
25	1523	0.91	1.39	1522	0.47	0.81
28	1639	−0.51	−0.20	1644	−0.69	−0.16
**Mode**	**PBE0**					
	**Cytosine**			**Cytosine·6H_2_O**		
	***ω***	**Δ1**	**Δ2**	***ω***	**Δ1**	**Δ2**
21	1265	−0.71	−0.32	1290	0.66	0.34
22	1323	0.40	0.58	1346	0.52	0.63
23	1400	0.47	0.19	1437	−0.49	−0.36
24	1457	−0.23	−0.60	1487	0.31	0.38
25	1520	−1.00	−0.90	1522	0.45	0.89
28	1644	0.60	−0.36	1640	−0.62	0.10

## Data Availability

The data presented in this study are available on request from the corresponding author.

## Supplementary Materials

The following supporting information can be downloaded at: https://www.mdpi.com/article/10.3390/molecules28052286/s1. Technical checks: a sketch of MOs and NTOs; absorption spectra in absolute intensities; additional results and checks on the vRR spectra; analysis of the normal modes in terms of internal coordinates. Cartesian coordinates of the optimized structures. Reference [84] is cited in the supplementary materials.
