# Supplementary material for: The Resonance Raman Spectrum of Cytosine in Water: Analysis of the Effect of Specific Solute–Solvent Interactions and Non-Adiabatic Couplings
Source: Molecules. 2023 Mar 1;28(5):2286. doi: 10.3390/molecules28052286 (PMC10005559; doi:10.3390/molecules28052286)
Supplement: Supplementary file 1 [file molecules-28-02286-s001.zip › molecules-2249488-supplementary.pdf]

Supporting Information:

The Resonance Raman Spectrum of Cytosine in water: Analysis of the Effect of Specific Solute-Solvent Interactions and Nonadiabatic Couplings

Qiushuang Xu [1,2,3], Yanli Liu [2], Meishan Wang [1,2] , Javier Cerezo [3,4] , Roberto Improta [5,*] and Fabrizio Santoro [3,*]

[1] School of Physics Engineering, Qufu Normal University, Qufu 2673100, China; qsxu1993@163.com (Q.X.); mswang1971@163.com (M.W.)
[2] School of Physics and Optoelectronics Engineering, Ludong University, Yantai 264025, China; yanliliu@ldu.edu.cn
[3] Consiglio Nazionale delle Ricerche, Istituto di Chimica dei Composti Organo Metallici (ICCOM-CNR), SS di Pisa, Area della Ricerca, via G. Moruzzi 1, I-56124 Pisa, Italy; javier.cerezo@uam.es
[4] Departamento de Química and Institute for Advanced Research in Chemical Sciences (IAdChem), Universidad Autónoma de Madrid, 28049 Madrid, Spain
[5] Consiglio Nazionale delle Ricerche, Istituto di Biostrutture e Bioimmagini (IBB-CNR), Via De Amicis 95, I-80145 Napoli, Italy
* Correspondence: roberto.improta@cnr.it (R.I.); fabrizio.santoro@pi.iccom.cnr.it (F.S.)

Contents

S1 Additional analysis of the excited electronic states at the FC position **S-3**

S1.1 Cytosine in PCM S-3

S1.2 Cluster Cytosine·6H_2O in PCM S-6

S2 Absorption spectra **S-8**

S3 Additional results for Vibrational Resonance Raman **S-11**

S3.1 Spectra computed with FC|VG *Int* and FC|VH *Int* models for Cytosine in PCM S-12

S3.2 Spectra computed with FC|VG *Int* and FC|VG *Sum* models for the cluster Cytosine·6H_2O in PCM S-13

S3.3 The effect of the inclusion of water molecules in the definition of the normal modes of Cytosine·6H_2O S-14

S3.4 Additional results and analysis with CAM-B3LYP S-16

S3.4.1 Spectrum in pre-resonance approximation S-16

S3.4.2 Analysis in terms of internal coordinates S-16

S3.4.3 Analysis of the vRR bands < 800 cm^{-1} and their enhancement in Cytosine·6H_2OS-21

S3.4.4 Raman excitation profiles S-21

S3.5 Additional results and analysis with PBE0 . . . . S-23

S3.5.1 Analysis of the modes involved in the most intense transitions for PBE0 . S-23

S3.5.2 Raman excitation profiles S-25

S4 Technical checks **S-26**

S4.1 FCclasses and ML-MCTDH deliver equivalent results if inter-states couplings are switched off S-26

References **S-28**

S1 Additional analysis of the excited electronic states at the FC position

In Tables 1 and 2 of main text, the excited electronic states are analysed and their character is assigned from the analysis of the Natural Transition Orbitals (NTOs). Such orbitals are reported here for Cytosine in PCM and Cytosine·$6H_2O$ in PCM, together with the weights of the corresponding transitions, and compared with the Kohn-Sham Molecular Orbitals (MOs). The main orbital transitions between MOs for each electronic state are also reported in Tables S1 for Cytosine and S2 for Cytosine·$6H_2O$, respectively.

S1.1 Cytosine in PCM

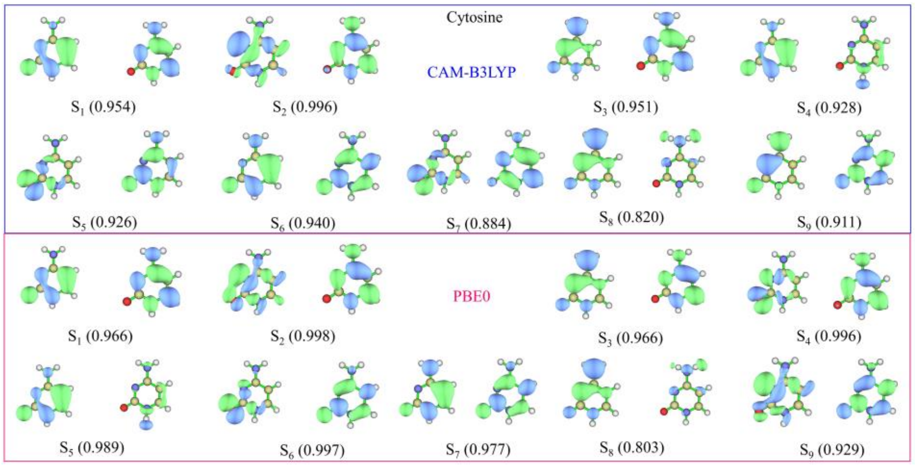

Figure S1: Natural transition orbitals (NTOs) in water at ground-state geometry of the first 9 excited states of Cytosine calculated with CAM-B3LYP (top) and PBE0 (bottom) at the GS geometry, plotted with an isovalue 0.04. The weight with which each transition contributes to the corresponding excited state is also reported.

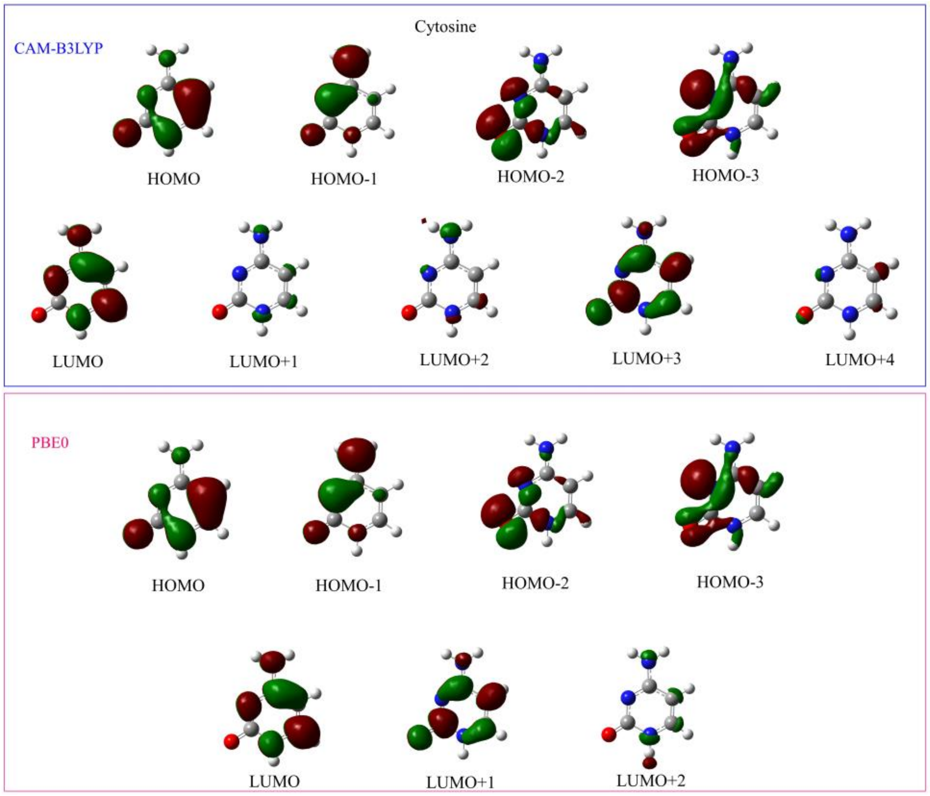

Figure S2: Molecular orbitals (MOs) of Cytosine in water calculated with CAM-B3LYP (top) and PBE0 (bottom) at the GS geometry, plotted with an isovalue 0.04.

Table S1: Symmetry, vertical excitation energy E_{gf} (eV), oscillator strength (δ_{OPA}) and main MOs transitions of the first 9 excited states of Cytosine in water (PCM), calculated with CAM-B3LYP and PBE0 with the 6-311+G(d,p) basis set.

STATE	CAM-B3LYP				
	Sym.	E_{gf}(eV)	δ_{OPA}	Trans.	Coeff.
S_1	A'	5.15	0.12	H→L	0.69
S_2	A"	5.70	0.0028	H-3→ L	0.55
				H-2→L	0.40
S_3	A'	5.94	0.21	H-1→L	0.68
S_4	A"	6.25	0.0054	H-1→L+1	0.66
S_5	A"	6.30	0.0007	H-2→L+3	0.55
S_6	A'	6.48	0.38	H→L+3	0.67
S_7	A"	6.57	0.0002	H-3→L	0.522
				H-2→L+3	0.33
S_8	A"	6.71	0.0038	H-1→L+1	0.53
				H→L+2	0.32
S_9	A'	6.89	0.40	H-1→L+3	0.40
**STATE**	**PBE0**				
	Sym.	E_{gf}(eV)	δ_{OPA}	Trans.	Coeff.
S_1	A'	4.98	0.092	H→L	0.68
S_2	A"	5.44	0.0025	H-3→L	0.53
				H-2→L	0.46
S_3	A'	5.65	0.15	H-1→L	0.68
S_4	A"	5.78	0.0002	H-3→L	0.52
				H-2→L	0.46
S_5	A"	6.13	0.0053	H→L+2	0.69
S_6	A"	6.17	0.0001	H-2→L+1	0.68
S_7	A'	6.30	0.21	H→L+1	0.64
S_8	A"	6.62	0.0028	H-1→L+2	0.59
S_9	A"	6.66	0.0025	H-3→L+1	0.67

S1.2 Cluster Cytosine$\cdot$6H_2O in PCM

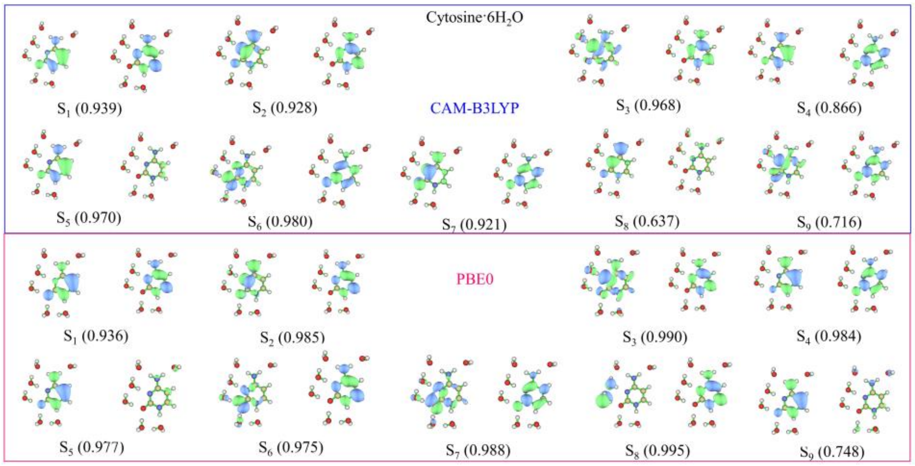

Figure S3: Natural transition orbitals (NTOs) in water at ground-state geometry of the first 9 excited states of Cytosine$\cdot$6H_2O calculated with CAM-B3LYP (top) and PBE0 (bottom) at the GS geometry, plotted with an isovalue 0.04. The weight with which each transition contributes to the corresponding excited state is also reported.

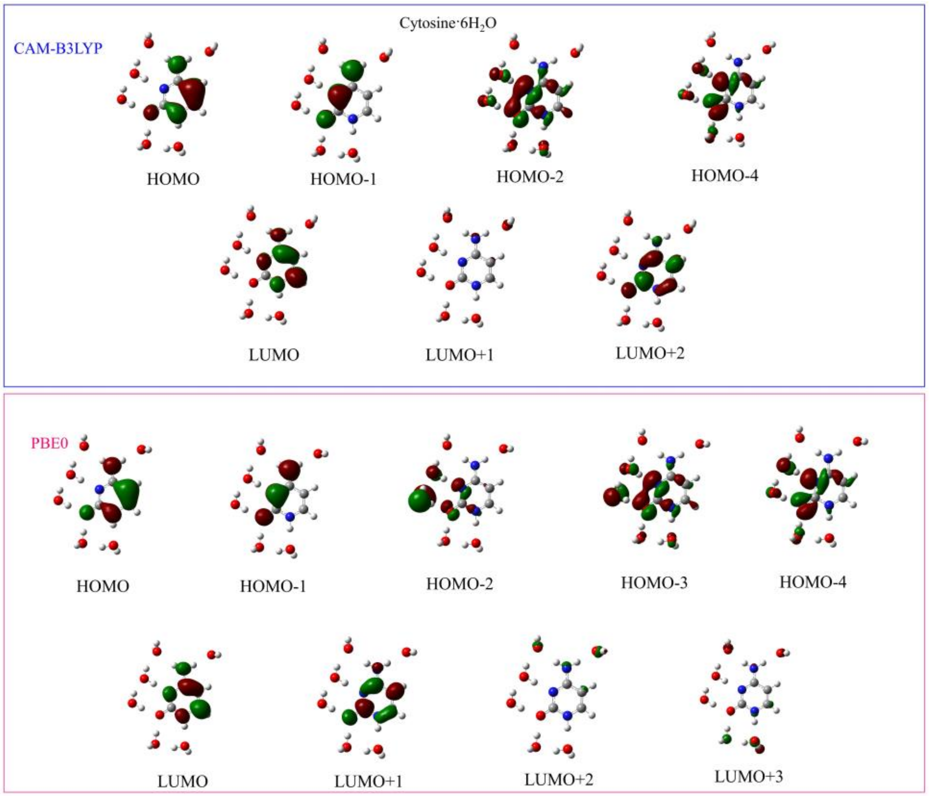

Figure S4: Molecular orbitals (MOs) of Cytosine in water calculated with CAM-B3LYP (top) and calculated with PBE0 (bottom) at the GS geometry, plottd with an isovalue 0.04.

Table S2: Vertical excitation energy E_{gf} (eV), oscillator strength (δ_{OPA}) and main MOs transitions of the first 9 excited states of the cluster Cytosine.6H_2O in water (PCM), calculated with CAM-B3LYP and PBE0 and the 6-311+G(d,p) basis set.

STATE	CAM-B3LYP			
	E_{gf}(eV)	δ_{OPA}	Trans.	Coeff.
S_1	5.25	0.19	H→L	0.67
S_2	5.98	0.15	H-1→L	0.59
S_3	6.07	0.028	H-2→L	0.53
S_4	6.31	0.27	H→L+2	0.65
S_5	6.47	0.0054	H→L+1	0.65
S_6	6.71	0.0043	H-2→L+2	0.59
S_7	6.84	0.54	H-1→L+2	0.65
S_8	6.96	0.0075	H-1→L+1	0.51
S_9	6.97	0.025	H-4→L+2	0.48
STATE	PBE0			
	E_{gf}(eV)	δ_{OPA}	Trans.	Coeff.
S_1	5.11	0.15	H→L	0.66
S_2	5.69	0.11	H-1→L	0.63
S_3	5.78	0.022	H-3→L	0.50
S_4	6.08	0.16	H→L+1	0.68
S_5	6.22	0.0028	H→L+2	0.68
S_6	6.26	0.0001	H-4→L	0.60
S_7	6.48	0.0013	H-3→L	0.57
S_8	6.54	0.0048	H-2→L	0.61
S_9	6.64	0.0054	H→L+3	0.60

S2 Absorption spectra

Figure S5 shows that the general shape of the computed absorption (ABS) spectra, compared with experiment in Figure 2 in the main text as normalized intensities, nicely agree even in absolute intensities with the large-window spectrum of ref.[S1] (the other experimental spectrum is given in arbitrary units, see[S2]). Turning to a more detailed analysis of the position of the bands, it should be noticed that a shift of ~ 0.08-0.1 eV seems to exist between the maxima of the first band of the two experimental spectra.[S1,S2] Such discrepancy is possibly caused by the fact that spectra were digitalized from the figures of the corresponding papers and, since the spectrum in ref[S1] covers a much larger energy window, from ~ 2 to 10 eV, the digitalization is less precise. Computed VG and LVC spectra have been red-shifted by 0.45 eV (CAM-B3LYP) or 0.3 eV (PBE0), in order to match the position of the maximum of the lowest energy experimental band as reported in ref.[S2] This result is consistent with the data in Table S1. The shapes of the spectra computed with different methods in Figure S5 (and Figure 2 in the main text) are similar up to 4.75 eV (exp. values). However, describing the solvent with PCM only, the absorption intensity in the valley at 4.75~5.0 eV appears to be overestimated both with PBE0 and (at a slighlty lower extent) with CAM-B3LYP. For energies >5.0 eV, the spectra for both functionals are red-shifted comparing with the experimental spectra,[S1] evidencing an underestimation of the energy difference of higher energy states with respect to the lowest one. CAM-B3LYP performs better than PBE0 on the simulation of the relative intensities of the experimental second and third peaks.

The nonadiabatic spectra calculated with LVC model (CAM-B3LYP) exhibit a very weak blue-shift of the highest energy peak. However, on balance, LVC results of both functionals are very similar to corresponding FC|VG results, showing that the effect of inter-state couplings on the ABS spectra is only moderate (whereas for vRR, at least in the high energy-wing of the range we explored are much more remarkable, see main text).

Computations on the Cytosine· $6H_2O$ cluster shows that inclusion of specific solute/solvent interactions remarkably improves the relative intensity of the valley at 4.75~5.0 eV. At higher

energies the PBE0 prediction worsen, since the small energy gap and the relative intensities of $\pi\pi_2^*$ and $\pi\pi_3^*$ lead to a huge underestimation of the intensity of high-energy spectrum. AS discussed in the main text, this is due to the effect of intruder CT states and the necessity to include more states in the computations. On the contrary, CAM-B3LYP spectrum matches nicely the experimental shape.

Interestingly the inclusion of specific solute-solvent interactions causes a blue-shift of the entire spectrum, increasing the computational error on its position by $\sim$ 0.2 eV.

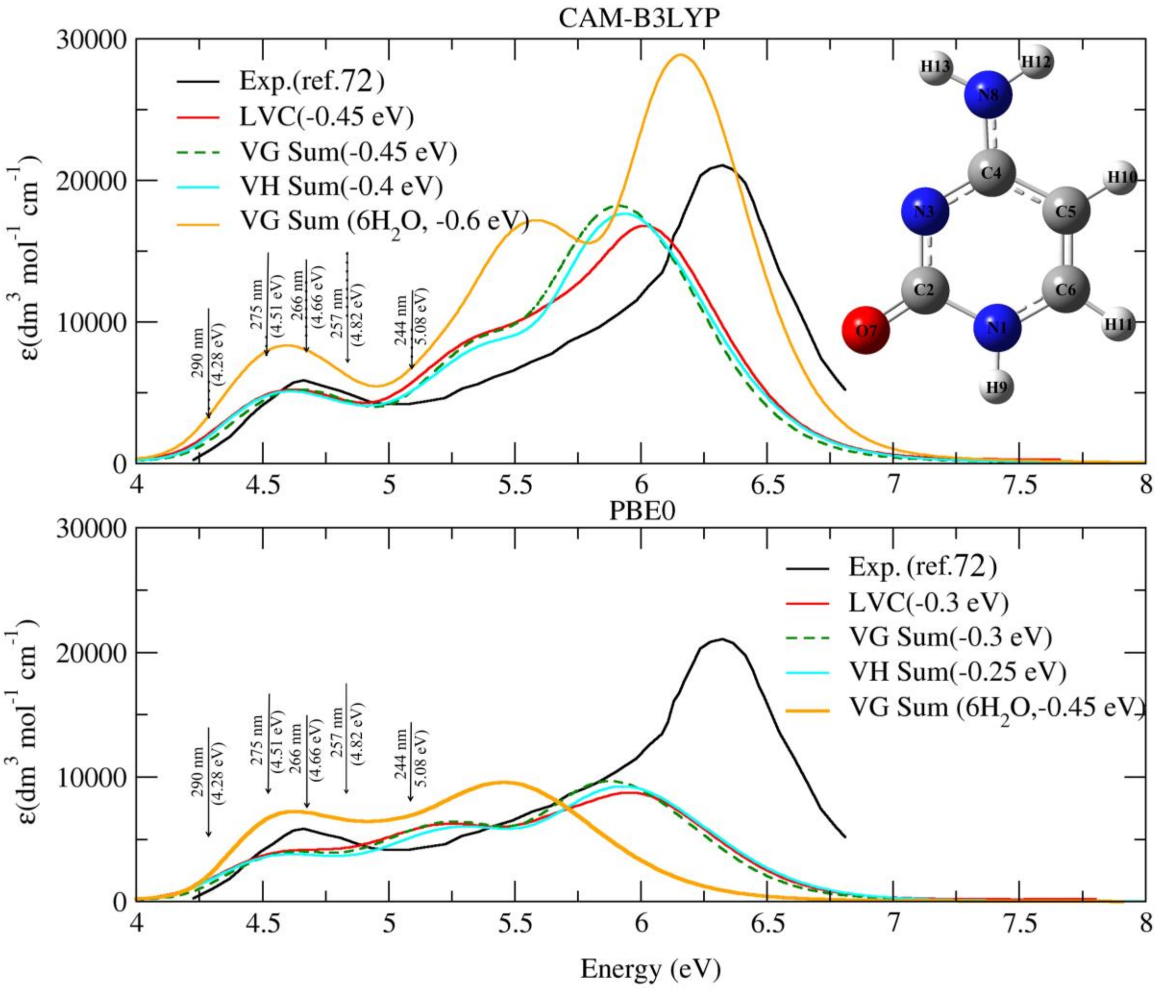

Figure S5: Absorption spectra of Cytosine computed by by the LVC model,FC|VG, FC|VH and FC|VG which considering the 6H_2O effect, convoluted with a Gaussian of HWHM = 0.12 eV and Lorentzian of HWHM = 0.04 eV. Experimental data, in water, from ref.[S3] Arrows indicate the excitation wavelength used in the vRR experiments in[S2] .

VH calculations are also shown in Figure S5. FC|VH and FC|VG spectra are quite similar, except for a general blueshift of the FC|VH spectra, because of the zero-point energy differences in the ground and electronic states. Table S3 reports the imaginary frequencies detected in the excited states when adopting VH model. In order to complete the computations, they were simply turned to real. This is clearly an arbitrary choice. Therefore, the adopted procedure rises some doubts on the reliability of the small shift of the VH spectrum with respect to the VG one (since turning the imaginary frequencies to real, potentially also displaces the position of the 0-0 transition and therefore of the whole spectrum.

On the contrary, the fact that VG and VH spectra have similar shapes seems to indicated that quadratic terms do not have a large effect. The existence of imaginary frequencies suggest that a more correct computation of the spectra beyond the VG model, would require anharmonic computations, an extremely challenging task. Even this solution would probably be in principle incomplete, because several of these imaginary frequencies may be due to inter-state couplings. On the other hand, the effect of the latter couplings has been investigated without including quadratic couplings by comparing VG and LVC results (Figure 1) and they were found to be limited.

Table S3: Excited-states normal modes carrying imaginary frequencies. Only relevant for VH calculations

State	CAM-B3LYP	PBE0
S_1	410.6i,348.1i,297.8i,74.5i	428.3i,354.2i,296.3i,46.7i
S_2	613.8i,388.3i,217.8i	924.5i,601.3i,344.2i,188.5i
S_3	451.7i,280.3i,140.9i	596.2i,320.3i,180.0i
S_4	2102.1i,695.9i	474.8i,441.9i,239.1i
S_5	1706.5i,801.4i,392.7i,341.1i	767.2i
S_6	579.9i,303.4i,243.9i	878.3i,549.5i,350.1i,219.0i
S_7	1383.5i,310.5i ,211.2i,73.9	489.0i,309.9i,156.8i,51.2i
S_8	517.2i	1763.8i,1194.4i,443.0i,42.3i

S3 Additional results for Vibrational Resonance Raman

Figure S6 shows that both computed and experimental spectra at 275 and 266 nm are very similar to those analysed in the main text at 290 and 257 nm.

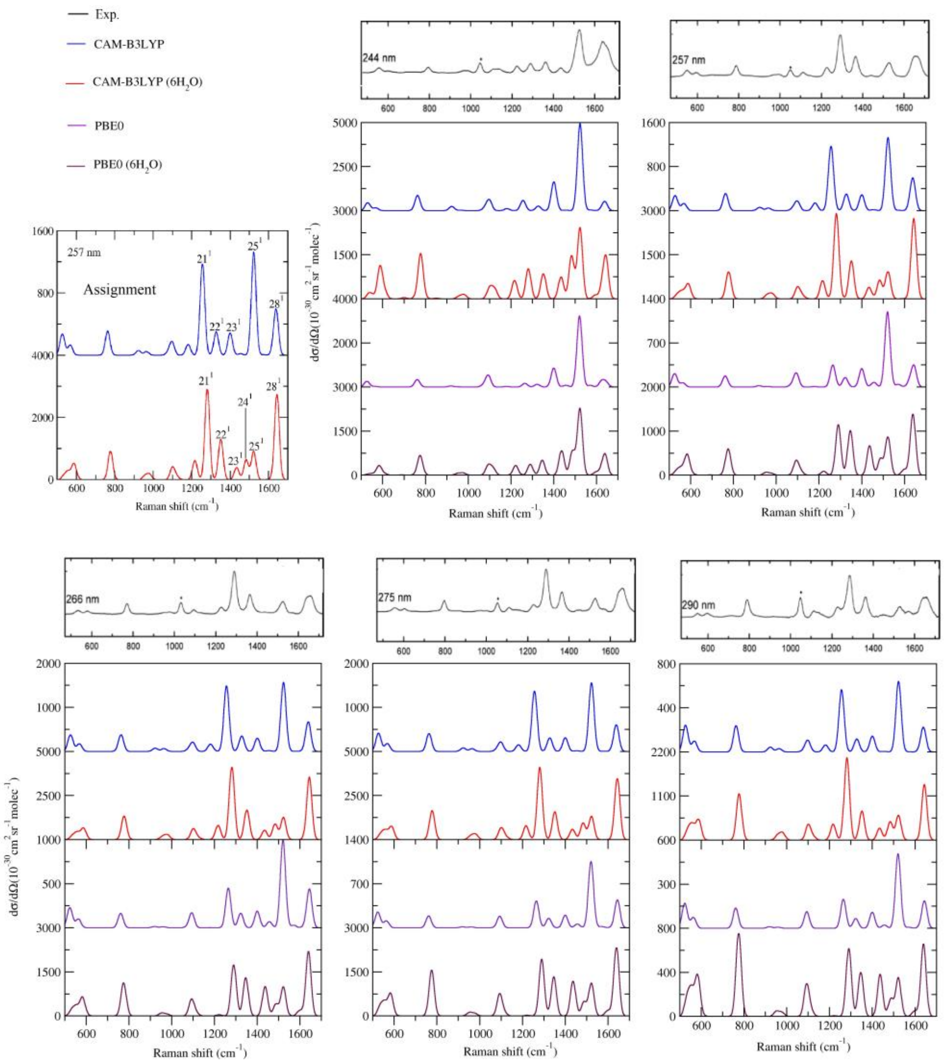

Figure S6: Vibrational resonance Raman spectra computed by VG *Int* in water for Cytosine in PCM and Cytosine·$6H_2O$ in PCM, convoluted with a Lorentzian with damping $\gamma = 0.04$ eV and a Gaussian of HWHM = 0.12 eV, calculated with CAM-B3LYP and the 6-311G+(d,p) basis set. On the top of each panel, we report the experimental data in aqueous solutions, reprinted with permission from.[S2] Copyright 2007 American Chemical Society. The experimental band marked with an asterisk is attributed to the internal standard and experimental spectra have been scaled to the height of the largest peak in each spectrum and off-set along the ordinate for clarity.

S3.1 Spectra computed with FC|VG *Int* and FC|VH *Int* models for Cytosine in PCM

Figure S7 shows that VH spectra are very similar to VG ones.

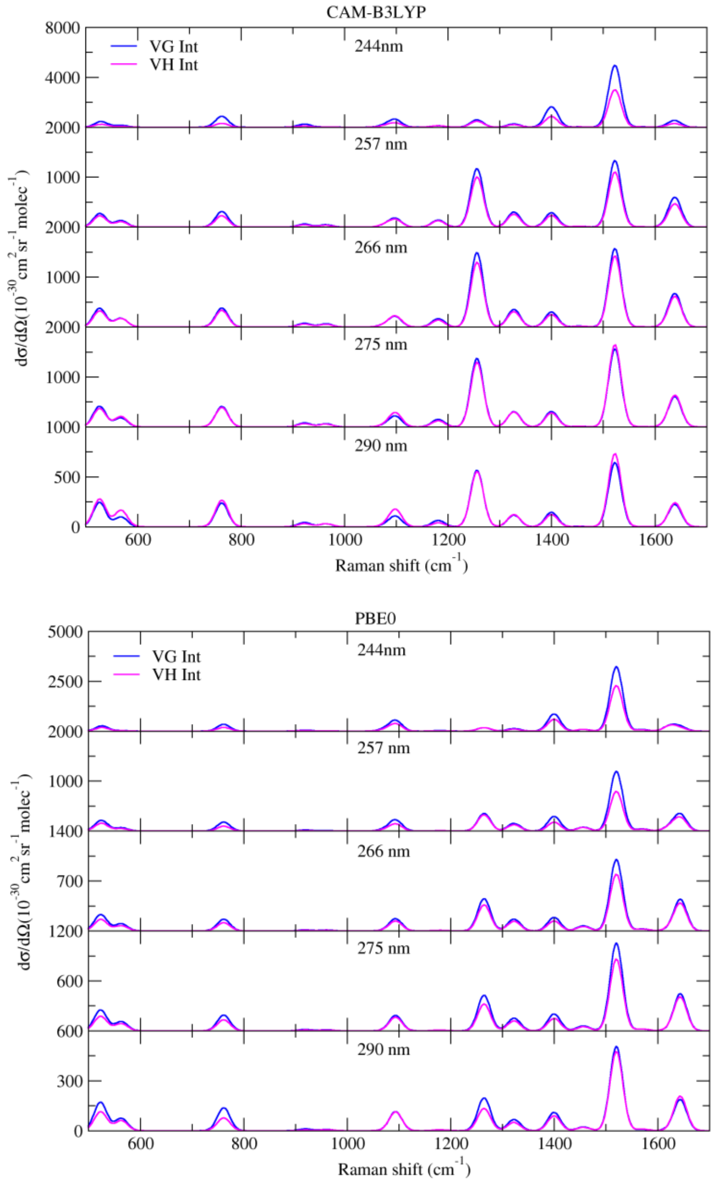

Figure S7: Vibrational resonance Raman spectra of Cytosine computed by the VG *Int* and VH *Int* levels convoluted with a Lorentzian with damping $\gamma = 0.04$ eV and a Gaussian of HWHM = 0.12 eV, calculated with CAM-B3LYP and PBE0 with 6-311G+(d,p) basis sets in water.

S3.2 Spectra computed with FC|VG *Int* and FC|VG *Sum* models for the cluster Cytosine·6H_2O in PCM

Like Figure 3 in the main text for Cytosine in PCM, Figure S8 shows that even for the cluster Cytosine·6H_2O in PCM, interferential effects are moderate, since *Int* and *Sum* spectra are quite similar.

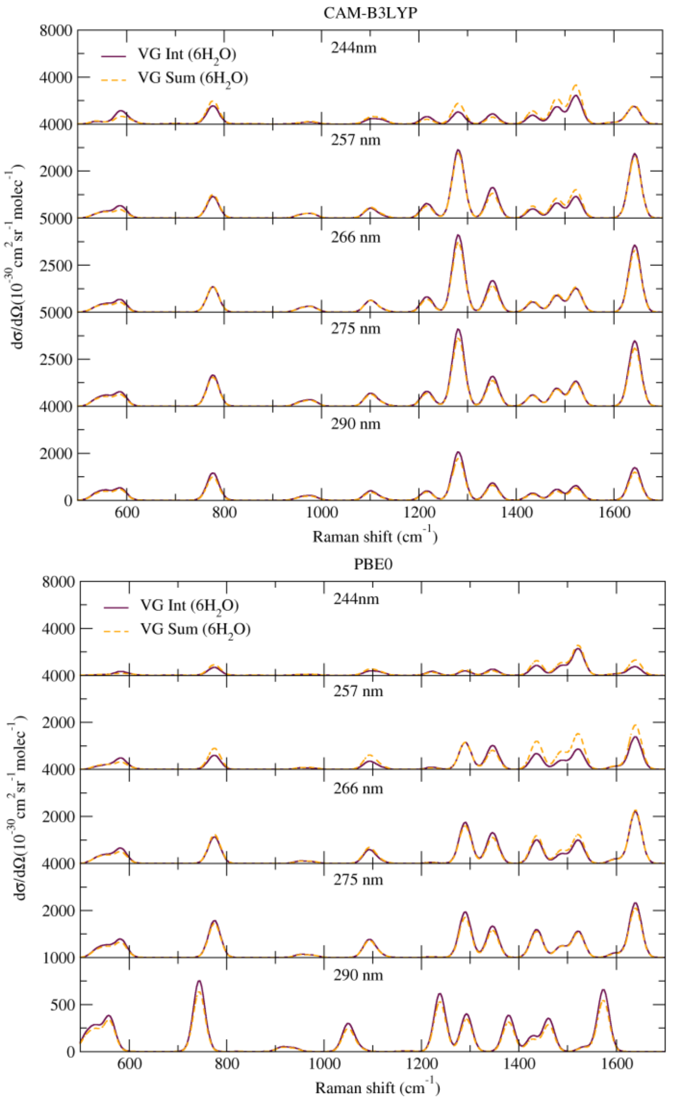

Figure S8: Vibrational resonance Raman spectra of Cytosine computed by FC|VG *Int* and FC|VG *Sum* levels which considering the 6H_2O effect, convoluted with a Lorentzian with damping γ = 0.04 eV and a Gaussian of HWHM = 0.12 eV, calculated with CAM-B3LYP and PBE0 with 6-311G+(d,p) basis sets in water.

S3.3 The effect of the inclusion of water molecules in the definition of the normal modes of Cytosine·6H_2O

Figure S9 reports a sketch of the normal modes corresponding to the most intense vRR fundamental bands computed with CAM-B3LYP for the Cytosine and for the cluster Cytosine·6H_2O without removing the components of the Hessian corresponding to the 6 water molecules. Comparison with the analogous Figure 5 of the main text, shows that even allowing the water molecules to contribute to the normal modes, the shape of those relevant for vRR changes very slightly.

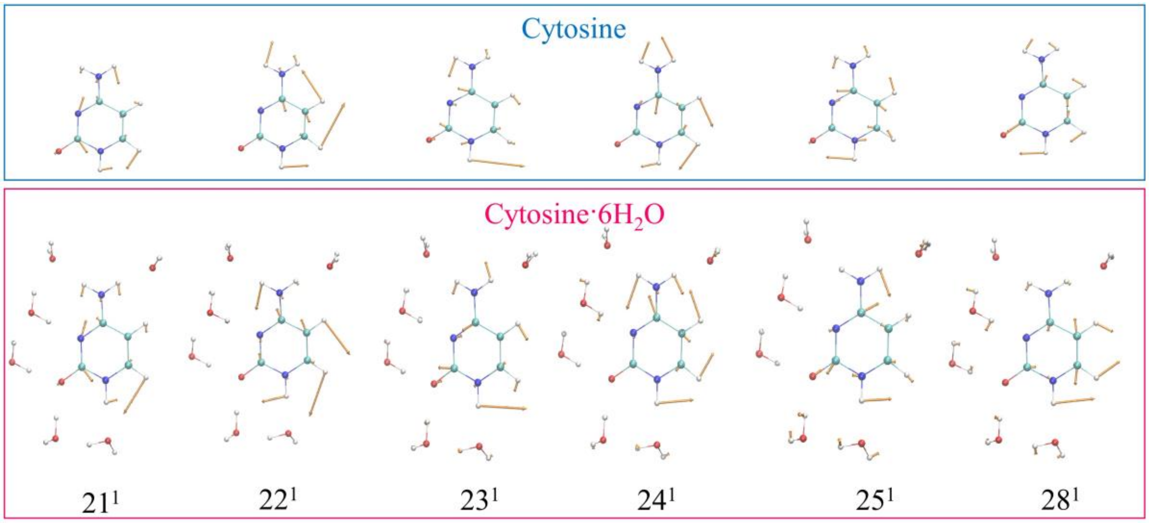

Figure S9: Schematic representation of the most relevant vRR-active vibrational modes of Cytosine and Cytosine·6H_2O, calculated with CAM-B3LYP and the 6-311G+(d,p) basis set in water (PCM).

Figure S10 shows that for the cluster Cytosine·6H_2O computations with the two different strategies, including the water molecules in the definition of the normal modes (”include”) or not (as done in the main text) deliver quite similar results.

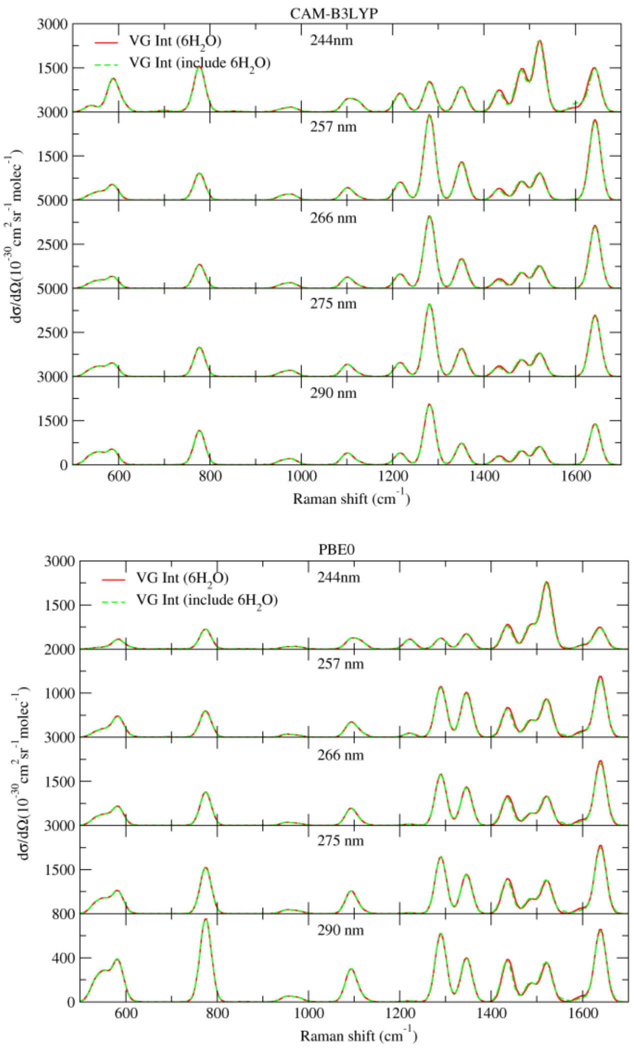

Figure S10: Vibrational resonance Raman spectra of Cytosine 6H_2O in water (PCM) comptued with the FC|VG *Int* model and convoluted with a Lorentzian with damping $\gamma = 0.04$ eV and a Gaussian of HWHM = 0.12 eV, calculated with CAM-B3LYP and the 6-311G+(d,p) basis set. The spectra analysed in the main text (red lines) computed by defining normal modes strictly localized on the Cytosine (i.e. removing the components of the Hessian corresponding to the coordinates of the water molecules), are compared with the spectra computed defining normal modes on the whole cluster (by diagonalization of the full Hessian matrix).

S3.4 Additional results and analysis with CAM-B3LYP

S3.4.1 Spectrum in pre-resonance approximation

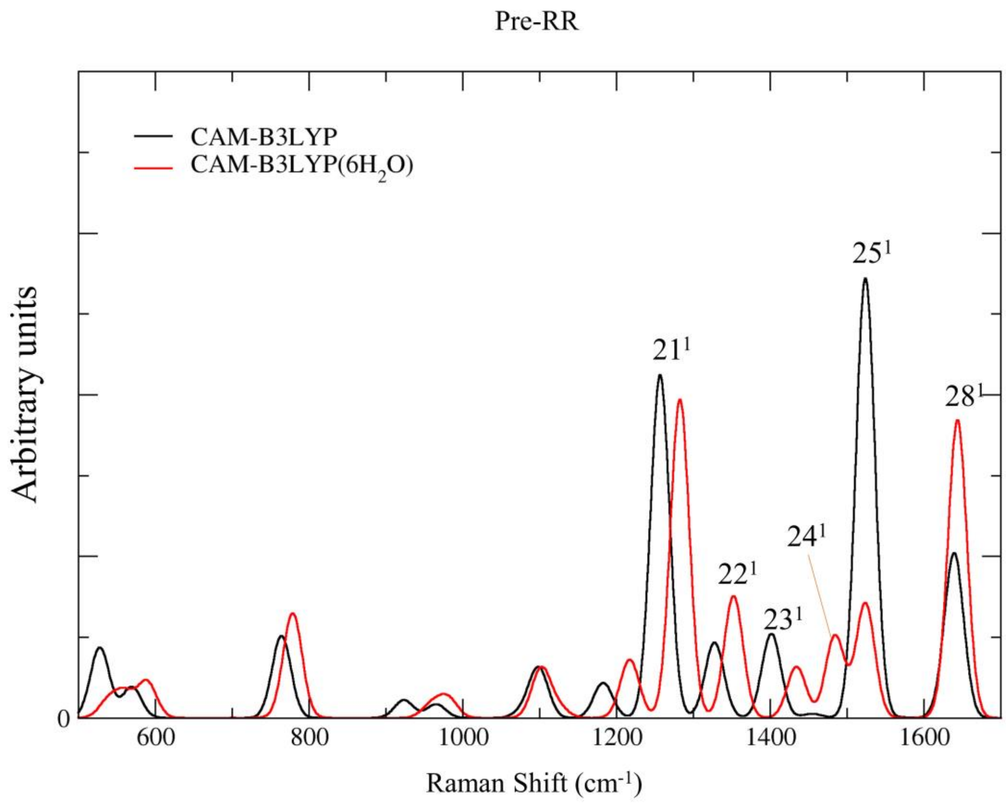

Figure S11: Vibrational resonance Raman spectra of Cytosine computed in the pre-resonance regime in water and which considering the $6H_2O$ effect, broadened with a Gaussian of HWHM = 15 cm⁻1. CAM-B3LYP/6-311G+(d,p) computations with PCM.

S3.4.2 Analysis in terms of internal coordinates

In order to get a deeper understanding of the effect of the specific interaction with the 6 water molecules on the vRR intensity, we analysed the composition of the normal modes in terms of internal coordinates. Some caveats are, however, necessary. The choice of internal coordinates is not univocal, and it is further complicated by the transformation from redundant to non-

redundant internal coordinates. Moreover, the contributions of the different internal coordinates to the normal modes are usually small, especially when redundant sets of internal coordinates are used, indicating that all modes are combinations of displacements along several internal coordinates (see Table S4). The mixed natures of internal coordinates (which usually include bond distances, angles and dihedrals) further complicate the interpretation of the relative values of the coefficients describing the contribution of different internal coordinates to a given normal mode. Finally, it should be stressed that the assignment based on the columns of the normal mode matrix, $\mathbf{L}$, or on the rows of its (generalized) inverse, $\mathbf{L}^{-1}$, is not coincident, given the non-orthogonality of these coordinates. This analysis, therefore, provides a rather qualitative description, which however can still be useful to individuate the major contributions of internal coordinates to a given normal mode.

In this analysis, the contribution of internal coordinates to the modes is computed from the elements in the columns of the normal mode matrix, $\mathbf{L}$, where $\Delta\mathbf{S} = \mathbf{LQ}$. The relative contribution of internal coordinates S_j in mode Q_k is then computed as,

$$Contr.[S_j \ in \ Q_k](\%) = \frac{|L_{jk}|}{\sum_i |L_{ik}|} \times 100 \qquad \text{(S1)}$$

In order to reduce the number of contributions and simplify the description of the modes, the set of internal coordinates used in this analysis corresponds to those of the Z-matrix plus bond distances that are missing from the Z-matrix when rings are present.

On the one hand, the displaced geometries of Cytosine including or not including the 6 water molecules in terms of internal valence coordinates are quite similar (Table S5). This finding suggests that the differences in the vRR spectra induced by the 6 water molecules are not (or not only) due to differences in the equilibrium positions. On the other hand, although the relevant normal modes are localized on the Cytosine, i.e. they do not involve the movement of the water molecules, it is noted that their composition in terms of internal coordinates of the Cytosine does change. Therefore the (approximately) same displacement in terms of internal coordinates

may translate into different displacements along the normal modes explaining the different vRR intensities.

Mode 21, corresponding to the strong 21^1 band, is an in-plane distortion made up of both the C2N3, C2N1 stretches and C5C6H11, N1C6H11 bendings. Their weights for Cytosine in PCM, or considering the 6H_2O effects are similar, leading to a similar predicted intensity. On the contrary, the larger contribution of C4C5 stretch to mode 24 predicted when introducing the 6H_2O effects is probably what leads to a larger displacement and a more intense band. Also, mode 25 has a contribution of C4C5 stretching combined with C4N3 stretches and C6N1H9 bending. In this case, however, the contribution of C4C5 stretch is significantly smaller when accounting for the specific solute-solvent interactions and, likely for this reason, its intensity decreases. Conversely, the contribution of C6C5 stretching (undergoing a significant displacement) to mode 28 increases, accounting for the effect of the 6 H_2O, and this is probably connected to the enhancement of the intensity of the $\sim$1650 cm^{-1} band with the cluster model that improves the agreement with experiment.

Table S4: Analysis of the modes responsible for the strongest vRR fundamental bands in resonance with S1 electronic state. Frequencies (ω) scaled by 0.96 are in wavenumbers and shifts in atomic units.

Mode	Cytosine					Cytosine·6H_2O				
	ω	Δ1	Δ2	Assignment	Contr.	ω	Δ1	Δ2	Assignment	Contr.
21	1256	0.98	0.27	*st* (C2N3)	19	1281	-0.92	-0.42	*st* (C2N3)	18
				st (C2N1)	12				*st* (C2N1)	11
				bnd (C5C6H11)	10				*bnd* (N1C6H11)	13
				st (C4N3)	8				*st* (C4N3)	10
				st (C4N8)	7				*st* (C4N8)	7
				st (C6N1)	5				*st* (C6N1)	4
				st (C2O7)	3				*st* (C2O7)	4
				bnd (C6N1H9)	6				*bnd* (C6N1H9)	4
22	1327	-0.43	0.13	*bnd* (C5C6H11)	17	1351	0.54	-0.15	*bnd* (N16H11)	12
				bnd (C6C5H10)	17				*bnd* (C6C5H10)	17
				st (C4N8)	11				*st* (C4N8)	9
				bnd (C6N1H9)	10				*bnd* (C6N1H9)	7
				st (C2O7)	3				*st* (C2O7)	2
				st (C6C5)	7				*st* (C6C5)	8
				st (C2N3)	5				*st* (C2N3)	8
23	1400	-0.43	-0.80	*bnd* (C6N1H9)	20	1433	-0.33	-0.49	*bnd* (C6N1H9)	14
				st (C4N3)	9				*st* (C4N3)	12
				st (C2O7)	6				*st* (C2O7)	10
				bnd (C4N8H13)	10				*bnd* (C4N8H13)	8
24	1454	0.09	-0.07	*st* (C4N8)	12	1483	0.41	0.69	*st* (C4N8)	15
				st (C4C5)	6				*st* (C4C5)	15
				bnd (C6C5H10)	11				*bnd* (C6C5H10)	14
				bnd (C5C6H11)	12				*bnd* (N1C6H11)	11
				bnd (C6N1H9)	7				*bnd* (C6N1H9)	11
25	1523	0.91	1.39	*st*(C4C5)	12	1522	0.47	0.81	*st* (C4C5)	8
				bnd (C6N1H9)	11				*bnd* (C6N1H9)	10
				st (C4N3)	11				*st* (C4N3)	11
				st (C2O7)	0				*st* (C2O7)	10
				bnd (C6C5H10)	9				*bnd* (C6C5H10)	5
				st (C6N1)	8				*st* (C6N1)	6
28	1639	-0.51	-0.20	*st* (C2O7)	14	1644	-0.69	-0.16	*st* (C2O7)	5
				st (C6C5)	11				*st* (C6C5)	14
				bnd (C6N1H9)	10				*bnd* (C6N1H9)	13

[a] *st* and *bnd* stand for bond stretching and bending angle, respectively. “Contr.” indicates the relative contribution of the internal coordinate to the mode.

Table S5: The displacement (Δ,-Hs^{-1}.gs), which is the difference between the ground- and excited-state equilibrium geometries.

type	label	Cytosine	Cytosine·6H_2O	type	label	Cytosine	Cytosine·6H_2O
b	C6-C5	0.0732	0.0768	a	C6-N1-H9	1.340	-0.068
b	C6-N1	-0.0075	-0.0113	a	C2-N1-H9	-1.159	-0.898
b	C6-H11	-0.0040	-0.0043	a	C2-N3-C4	-2.435	-2.653
b	C2-N1	0.0356	0.0392	a	C4-C5-H10	-0.730	-0.527
b	C2-N3	-0.0376	-0.0262	a	C4-N8-H12	0.320	-0.026
b	C2-O7	0.0093	0.0007	a	C4-N8-H13	-0.357	-0.237
b	C4-C5	-0.0471	-0.0416	a	C5-C6-N1	-4.363	-4.672
b	C4-N3	0.0603	0.0497	a	C5-C6-H11	1.575	1.880
b	C4-N8	0.0261	0.0276	a	C5-C4-N3	-0.472	0.638
b	C5-H10	0.0021	0.0019	a	C5-C4-N8	3.270	2.095
b	N1-H9	0.0030	0.0077	a	N1-C6-H11	2.789	2.791
b	N8-H12	-0.0010	-0.0014	a	N1-C2-N3	3.712	2.696
b	N8-H13	-0.0028	-0.0016	a	N1-C2-O7	-5.213	-4.008
a	C6-C5-C4	3.739	3.019	a	N3-C2-O7	1.501	1.312
a	C6-C5-H10	-3.009	-2.492	a	N3-C4-N8	-2.798	-2.734
a	C6-N1-C2	-0.181	0.966	a	H12-N8-H13	0.037	0.193

bonds (b): Angstroms; bonding angles (a):degrees.

S3.4.3 Analysis of the vRR bands < 800 cm^{-1} and their enhancement in Cytosine·6H_2O

Table S6: Normal modes contributing to the most intense bands < 800 cm^{-1} computed at CAM-B3LYP/6-311G+(d,p) level with and without the explicit water molecules. Frequencies (ω), scaled by 0.96, are in wavenumbers, shifts in dimensionless coordinates.

Cytosine				Cytosine·6H_2O			
Mode	ω	Δ1	Δ2	Mode	ω	Δ1	Δ2
5	401.62	0	0	5	538.00	0.50	0.21
6	526.52	-1.03	-0.28	6	559.45	-0.57	-0.18
7	531.60	0.23	-0.55	7	587.94	0.72	-0.34
11	709.89	0	0	11	776.15	0.03	-0.32
12	763.15	0.78	0.73	12	777.57	-0.87	-0.64

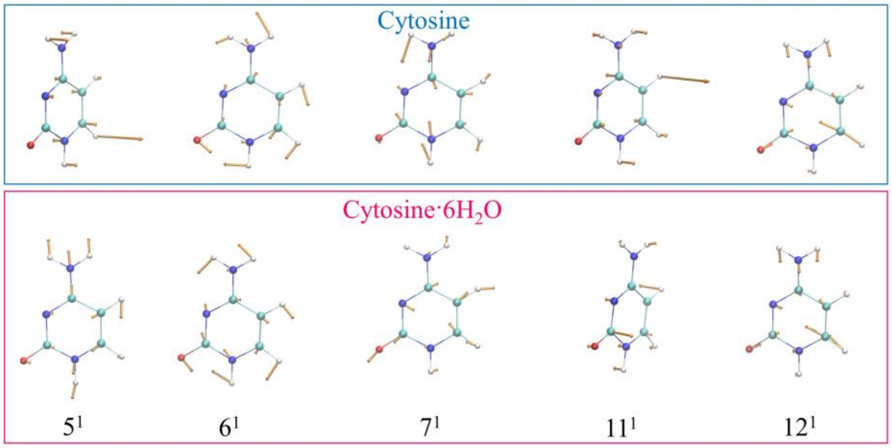

Figure S12: Schematic representation of the relevant vRR-active vibrational modes in the range of 500-800 cm^{-1} of Cytosine and Cytosine·6H_2O removing the components of the 6H_2O from the Hessian, calculated with CAM-B3LYP and the 6-311G+(d,p) basis set in water.

S3.4.4 Raman excitation profiles

Figures S13 compares the Raman excitation profiles obtained with VG *Int* model for the modes responsible for the bands at 600-800 cm^{-1}. They confirm that mode 5 and 11 are only activated in the cluster model, whereas they are vRR-inactive by symmetry considering Cytosine alone. For

the other modes, and in particular mode 7 differences are in any case quite remarkable. In all cases, the intensity above ∼ 5.5 eV is much larger for the cluster model. This finding is also confirmed for some of the higher-frequency modes in Figure S14 and is correlated to the larger intensity observed also in absorption (especially around 5.5 eV). The phenomenon is more evident in vRR than in ABS because the intensity of the former spectroscopy depend son the fourth power of the transition dipole, whereas the intensity of ABS depends on its second power. The figure also shows that nonadiabatic effects are much larger > 5.5 eV.

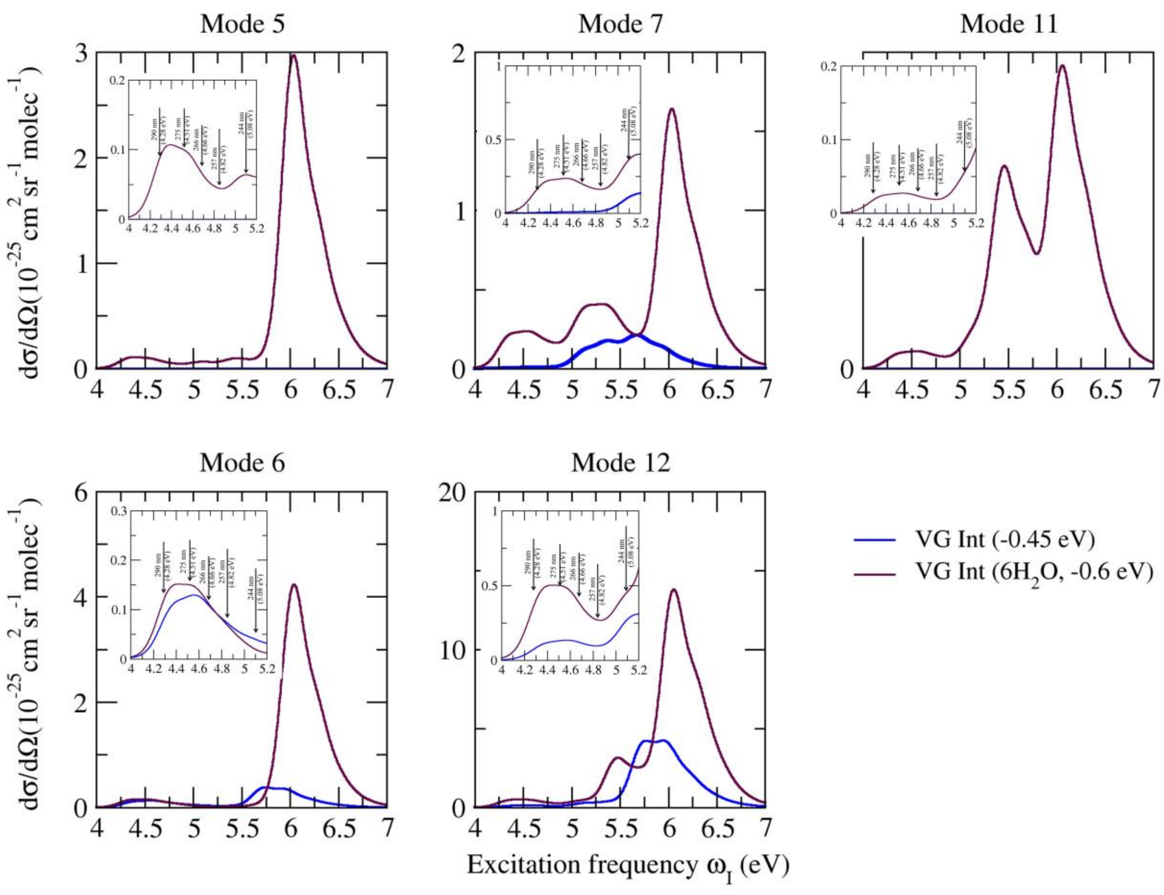

Figure S13: Raman excitation profiles of the five low-frequency modes relevant for the two bands at 600-800 cm^{-1}, at VG *Int* level for Cytosine and Cytosine 6H_2O, and convoluted with a Lorentzian with damping $\gamma = 0.04$ eV and a Gaussian of HWHM = 0.12 eV, calculated with CAM-B3LYP and the 6-311G+(d,p) basis set in water (PCM).

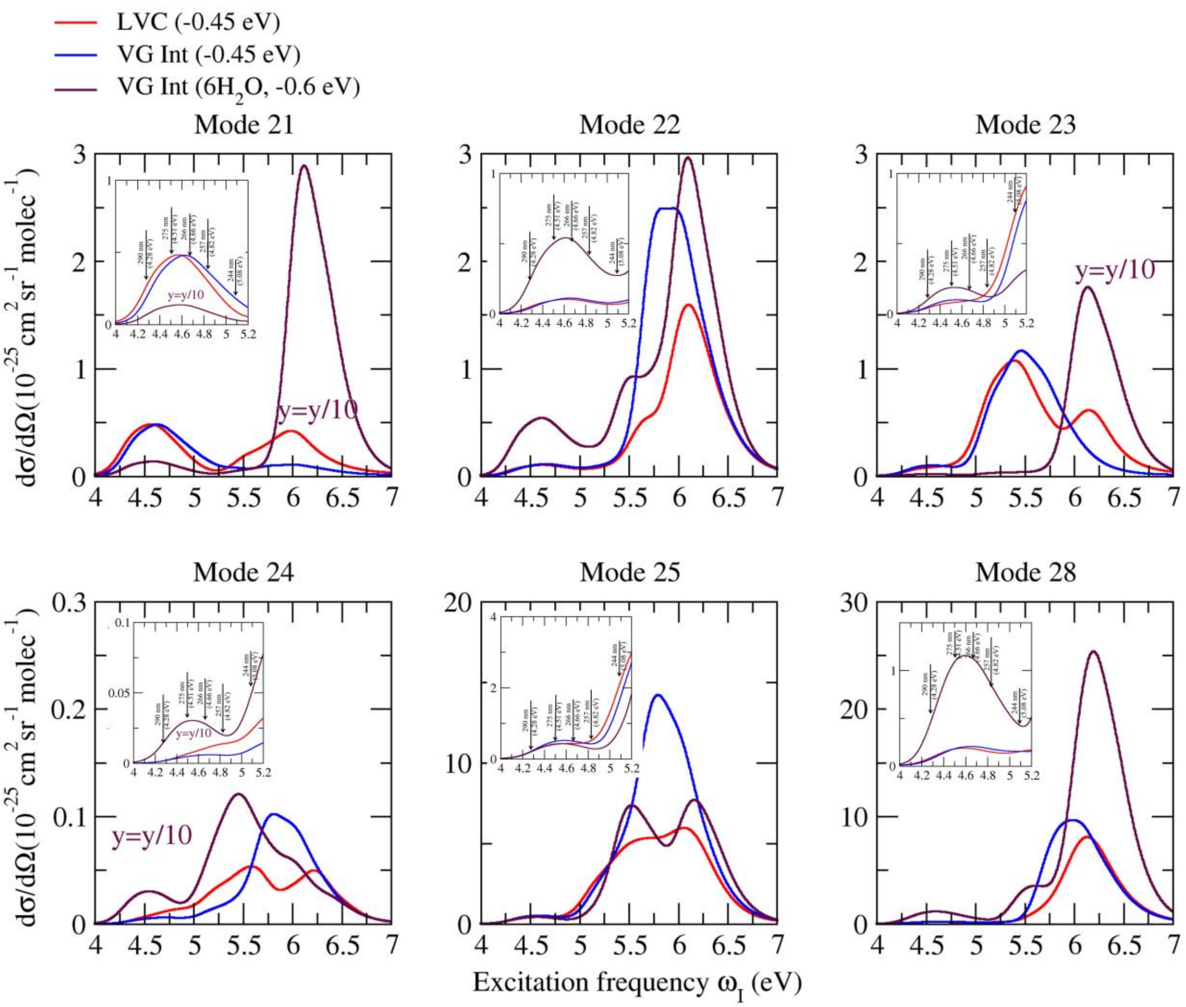

Figure S14: Raman excitation profiles of the six high-frequency modes with most intense vRR bands, computed at non-adiabatic LVC level and VG *Int* level for Cytosine, and at VG *Int* level for Cytosine $6H_2O$, convoluted with a Lorentzian with damping $\gamma = 0.04$ eV and a Gaussian of HWHM = 0.12 eV, calculated with CAM-B3LYP and the 6-311G+(d,p) basis set in water (PCM).

S3.5 Additional results and analysis with PBE0

S3.5.1 Analysis of the modes involved in the most intense transitions for PBE0

Table S7: Analysis of the modes responsible for the strongest vRR fundamental bands in resonance with S1 electronic state according to PBE0. Frequencies (ω) scaled by 0.96 are in wavenumbers and shifts in atomic units.

Mode	Cytosine					Cytosine·6H_2O				
	ω	$\Delta 1$	$\Delta 2$	Assignment	Contr.	ω	$\Delta 1$	$\Delta 2$	Assignment	Contr.
21	1265	-0.71	-0.32	*st* (C2N3)	19	1290	0.66	0.34	*st* (C2N3)	17
				st (C2N1)	13				*st* (N1C2)	12
				bnd (C5C6H11)	10				*bnd* (N1C6H11)	12
				st (C4N8)	8				*st* (C4N8)	9
22	1323	0.40	0.58	*bnd* (C5C6H11)	17	1346	0.52	0.63	*bnd* (N1C6H11)	11
				bnd (C6C5H10)	17				*bnd* (C6C5H10)	17
				bnd (C6N1H9)	10				*bnd* (C6N1H9)	7
				st (C4N8)	9				*st* (C4N8)	7
23	1400	0.47	0.19	*bnd*(C6N1H9)	21	1437	-0.49	-0.36	*bnd*(C6N1H9)	15
				st (C6N1)	10				*st* (C6-N1)	6
				bnd (C4N8H13)	10				*bnd* (C4N8H13)	8
				st (C2O7)	6				*st* (C2O7)	9
				st (C4C5)	10				*st* (C4C5)	87
24	1457	-0.23	-0.60	*st* (C4N8)	12	1487	0.31	0.38	*st* (C4N8)	15
				bnd (C5C6H11)	11				*bnd* (N1C6H11)	10
				bnd (C6C5H10)	10				*bnd* (C6C5H10)	12
				st (C4-C5)	6				*st* (C4-C5)	14
25	1520	-1.00	-0.90	*st*(C4C5)	12	1522	0.45	0.89	*st*(C4C5)	8
				bnd (C6N5H9)	9				*bnd* (C6N5H9)	12
				st (C4N3)	10				*st* (N3C4)	10
				bnd (C6C5H10)	9				*bnd* (C6C5H10)	5
				st (C2O7)	0				*st* (C2O7)	9
				bnd (C4N8H12)	6				*bnd* (C4N8H12)	8
28	1644	0.60	-0.36	*st*(C2O7)	19	1640	-0.62	0.10	*st*(C2O7)	7
				bnd (C6N1H9)	10				*bnd* (C6N1H9)	12
				st (C6C5)	5				*st* (C6C5)	12

[a] *st* and *bnd* stand for bond stretching and bending angle, respectively.

S3.5.2 Raman excitation profiles

Figure S15 confirms that also according to PBE0 nonadiabatic effects are much larger at > 5.5 eV. The high-energy wing of the Raman profiles is biased by the same lack of states in the computation (in particular S10) we discussed for absorption.

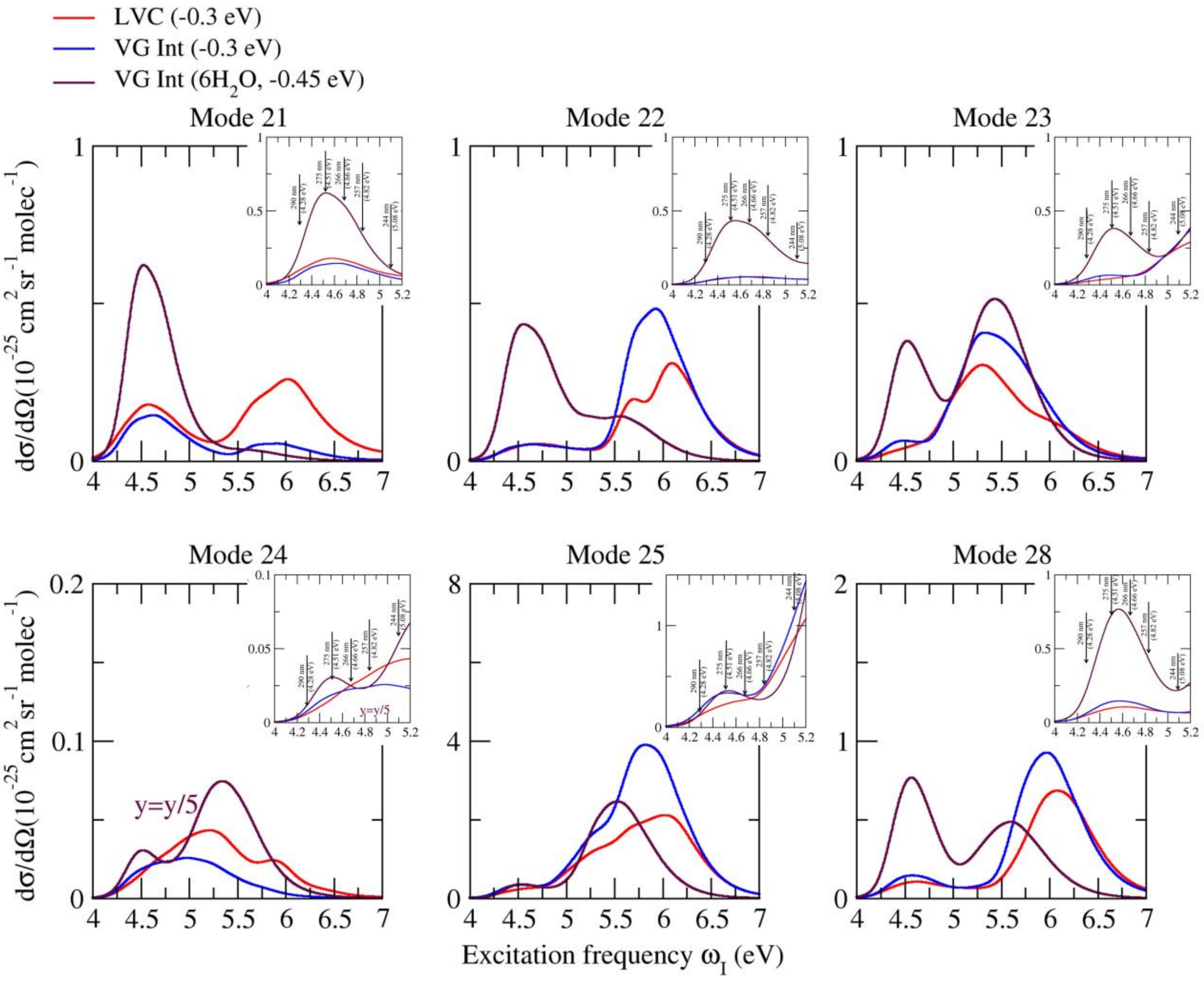

Figure S15: Raman excitation profiles for Cytosine, computed at non-adiabatic LVC level and with VG *Int*, and for Cytosine 6H_2O with VG *Int*, convoluted with a Lorentzian with damping $\gamma = 0.04$ eV and a Gaussian of HWHM = 0.12 eV, calculated with PBE0 and the 6-311G+(d,p) basis set in water.

S4 Technical checks

S4.1 FCclasses and ML-MCTDH deliver equivalent results if inter-states couplings are switched off

In this section we show that for cases in which the inter-state couplings are set to zero (”single state”), computations performed with analytical correlation functions by FCclasses3[S4] and with numerical wavepacket ML-MCTDH propagations by Quantics deliver practically indistinguishable results.

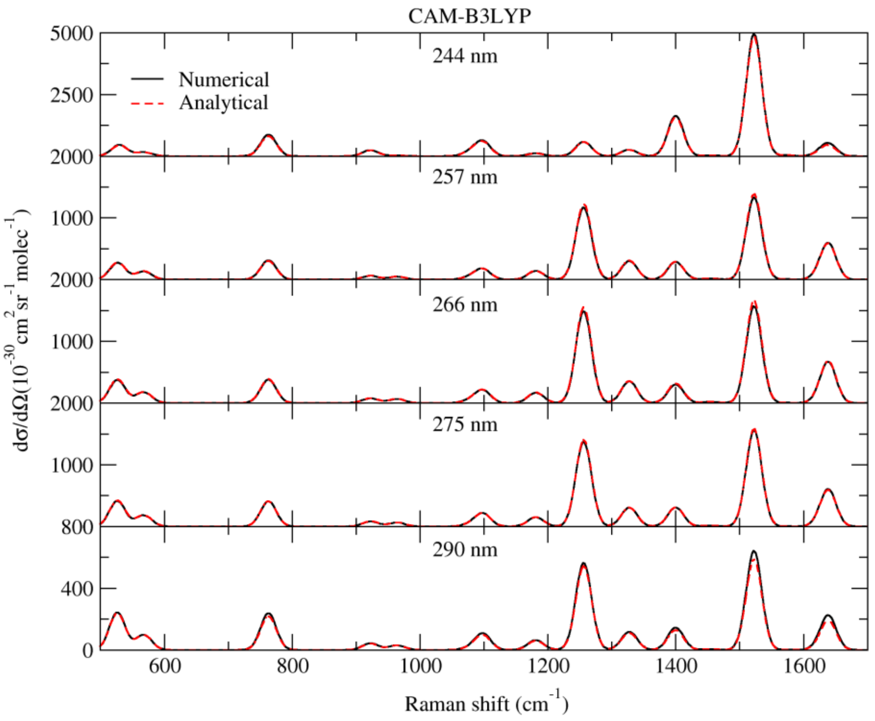

Figure S16: Comparison of the computations of the vibrational Resonance Raman spectrum of Cytosine with the model FC|VG *Int* (i.e. including interferences) obtained either with FCclasses and analytical correlation functions (”analytical”) or with numerical propagations with ML-MCTDH (”numerical”) with the LVC model setting the inter-state couplings to zero. Computations including the effect of the first eight states on the grounds of CAM-B3LYP/6-311G+(d,p) computations in water, with a damping $\gamma = 0.04$ eV.

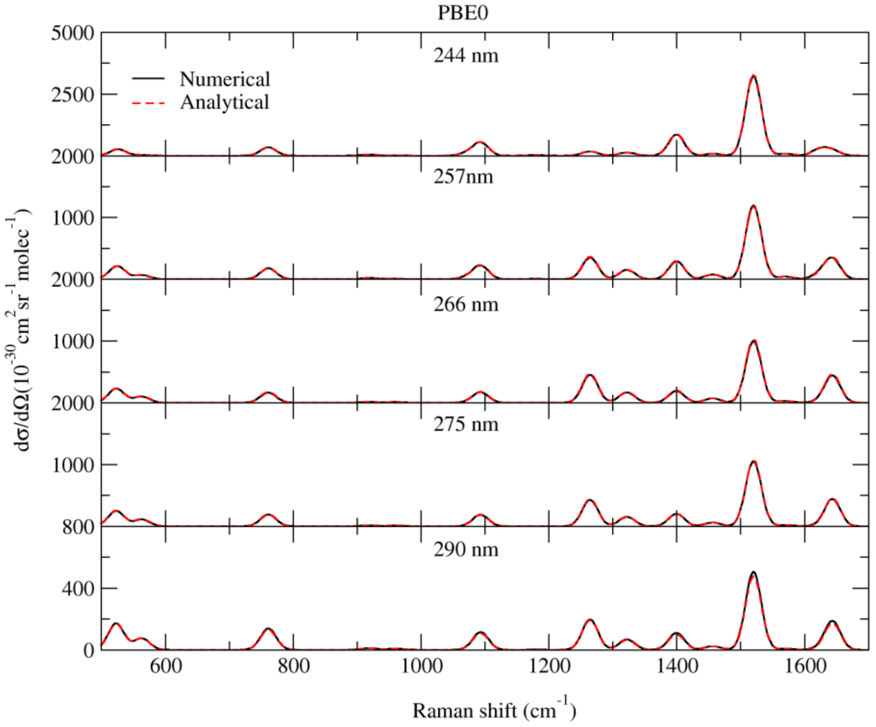

Figure S17: Comparison of the computations of the vibrational Resonance Raman spectrum of Cytosine with the model FC|VG *Int* (i.e. including interferences) obtained either with FC-classes and analytical correlation functions (”analytical”) or with numerical propagations with ML-MCTDH (”numerical”) with the LVC model setting the inter-state couplings to zero. Computations including the effect of the first eight states on the grounds of PBE0/6-311G+(d,p) computations in water, with a damping $\gamma = 0.04$ eV.

References

(S1) Zaloudek, F.; Novros, J. S.; Clark, L. B. The electronic spectrum of cytosine. *Journal of the American Chemical Society* **1985**, *107*, 7344–7351.

(S2) Billinghurst, B. E.; Loppnow, G. R. Excited-state structural dynamics of cytosine from resonance Raman spectroscopy. *The Journal of Physical Chemistry A* **2006**, *110*, 2353–2359.

(S3) Bazsó, G.; Tarczay, G.; Fogarasi, G.; Szalay, P. G. Tautomers of cytosine and their excited electronic states: a matrix isolation spectroscopic and quantum chemical study. *Physical Chemistry Chemical Physics* **2011**, *13*, 6799–6807.

(S4) Santoro, F.; Cerezo, J. *FCclasses*3, a code for vibronic calculations. Available at http://www.iccom.cnr.it/en/fcclasses. 2022, last accessed on February 2023.
